# Structural insights into the inhibition mechanism of human sterol *O*-acyltransferase 1 by a competitive inhibitor

**DOI:** 10.1038/s41467-020-16288-4

**Published:** 2020-05-18

**Authors:** Chengcheng Guan, Yange Niu, Si-Cong Chen, Yunlu Kang, Jing-Xiang Wu, Koji Nishi, Catherine C. Y. Chang, Ta-Yuan Chang, Tuoping Luo, Lei Chen

**Affiliations:** 1 State Key Laboratory of Membrane Biology, Institute of Molecular Medicine, Peking University, Beijing Key Laboratory of Cardiometabolic Molecular Medicine, 100871 Beijing, China; 2 Key Laboratory of Bioorganic Chemistry and Molecular Engineering, Ministry of Education and Beijing National Laboratory for Molecular Science, College of Chemistry and Molecular Engineering, Peking University, 100871 Beijing, China; 3 Department of Biochemistry and Cell Biology, Geisel School of Medicine at Dartmouth, Hanover, NH 03755 USA; 4 Peking-Tsinghua Center for Life Sciences, Peking University, 100871 Beijing, China; 5 Academy for Advanced Interdisciplinary Studies, Peking University, 100871 Beijing, China

**Keywords:** Transferases, Cryoelectron microscopy, Cardiovascular diseases, Endocrine system and metabolic diseases

## Abstract

Sterol *O*-acyltransferase 1 (SOAT1) is an endoplasmic reticulum (ER) resident, multi-transmembrane enzyme that belongs to the membrane-bound *O*-acyltransferase (MBOAT) family. It catalyzes the esterification of cholesterol to generate cholesteryl esters for cholesterol storage. SOAT1 is a target to treat several human diseases. However, its structure and mechanism remain elusive since its discovery. Here, we report the structure of human SOAT1 (hSOAT1) determined by cryo-EM. hSOAT1 is a tetramer consisted of a dimer of dimer. The structure of hSOAT1 dimer at 3.5 Å resolution reveals that a small molecule inhibitor CI-976 binds inside the catalytic chamber and blocks the accessibility of the active site residues H460, N421 and W420. Our results pave the way for future mechanistic study and rational drug design targeting hSOAT1 and other mammalian MBOAT family members.

## Abstract (web summary)

Sterol *O*-acyltransferase 1 (SOAT1, also named ACAT1) is an endoplasmic reticulum resident enzyme which catalyzes the esterification of cholesterol to generate cholesteryl esters. Here, authors report cryo-EM structures of human SOAT1 which reveal the binding site of the competitive inhibitor CI-976.

## Introduction

Cholesterol is an essential lipid molecule in the cell membranes of all vertebrate. It is important for maintaining the fluidity and integrity of the membrane and is the precursor for the biosynthesis of other crucial endogenous molecules, such as steroid hormones and bile acids. In addition, cholesterol can modulate the activity of many membrane proteins such as GPCR^1^ and ion channels^2^. The concentration of cellular free cholesterol is highly regulated^3^. Excessive intracellular cholesterol may form cholesteryl esters, which are catalyzed by the enzyme, sterol *O*-acyltransferase (SOAT), also called acyl-coenzyme A: cholesterol acyltransferase (ACAT). SOAT catalyzes the reaction between long chain fatty acyl-CoA and intracellular cholesterol to form the more hydrophobic cholesteryl ester, which is then stored in lipid droplets within the cell or transported in secreted lipoprotein particles to other tissues that need cholesterol. In addition to cholesterol, SOAT can use multiple sterols as substrates and activators^4^. Because of its functional importance, SOAT1 is a potential drug target for Alzheimer’s disease^5^, atherosclerosis^6^ and several types of cancers^7–10^.

Previous studies have shown that SOAT1 is an ER-localized multi-transmembrane protein that is evolutionary conserved from yeast to humans^11^. There are two SOAT enzymes in mammals: SOAT1 and SOAT2, which have a protein sequence identity of 48% in human (258 out of 537 residues aligned). SOAT1 is ubiquitously expressed in many types of cells^12^; while SOAT2 is mainly expressed in the small intestine and liver^13^. Due to the pathophysiological importance of SOAT, many SOAT inhibitors of various structural types have been made. The small molecule CI-976 is a prototypical member of SOAT inhibitors that belong to the fatty acyl amide analog family. Studies showed that feeding CI-976 reduced atherosclerotic plaques as well as reduced plasma cholesterol levels in animals fed with high cholesterol diet^14^. CI-976 exhibits competitive inhibition against fatty acyl-CoA^15,16^.

SOAT1 is the founding member of the membrane-bound *O*-acyltransferase (MBOAT) enzyme family, which transfers the acyl chain onto various substrates, including lipids, peptides and small proteins. There are 11 MBOAT family members in humans^17^, which participate in many physiological processes, such as the last step of triglyceride biosynthesis catalyzed by acyl-CoA: diacylglycerol acyltransferase 1 (DGAT1), the maturation of hedgehog morphogen catalyzed by hedgehog acyltransferase (HHAT), and the acylation of peptide hormone ghrelin catalyzed by ghrelin *O*-acyltransferase (GOAT). Recently, the X-ray crystal structure of DltB from *Streptococcus thermophilus* was determined and is the only available structure of an MBOAT family member^18^. The low-sequence conservation between DltB and SOAT (14.6% identity) hinders the accurate modeling of the human SOAT1 structure. Therefore, despite the important physiological functions of human SOAT enzymes, their architecture and mechanism remain elusive due to a lack of high-resolution structures. In this study, several human SOAT1 (hSOAT1) structures were determined. These structures reveal the architecture of hSOAT1 and the binding site of the competitive inhibitor CI-976, and provide a structural basis to understand the inhibition mechanism of these enzymes by small molecules.

## Results

### Structure determination

N-terminal GFP-tagged full-length (1–550) and N-terminal truncated hSOAT1 (66–550), solubilized in detergent micelles migrated before and after mouse TPC1 channel, which is a well-characterized dimeric channel with a molecular weight of 189 kDa (ref.^19^) (Supplementary Fig. 1a). This is consistent with previous studies that hSOAT1 is a tetramer with a molecular weight of around 260 kDa (ref.^20^) and that hSOAT1 became predominantly dimeric with the deletion of the N-terminal tetramerization domain^21^. We compared the recombinant SOAT1 enzyme activities in HEK293F suspension cell after infection by the recombinant BacMam viruses, versus those expressed in the stable transfectant of AC29 cell line^22^, or the adherent HEK293 cell^23^. We used the crude cell extracts solubilized by CHAPS as the enzyme source^22^, added the ^3^H-oleoyl-CoA and cholesterol present in taurocholate/phospholipid/cholesterol mixture as the substrates. The crude cell extracts from uninfected/untransfected HEK293 cells, or from AC29 cells (a CHO cell mutant devoid of endogenous SOAT1 protein^24^ were used as controls). The results (Supplementary Fig. 1b) showed that AC29 cells expressed essentially no SOAT1 activity; both the HEK293F cells and the adherent HEK293 monolayers expressed very low (endogenous) SOAT1 activity. Both the stable transfectant of AC29 cells and the stable transfectant of HEK293 cells expressed markedly higher SOAT1 activities, with the adherent HEK293 cells producing much higher recombinant SOAT1 activity than AC29 cells. These results are consistent with what were reported previously. Strikingly, HEK293F cells infected with SOAT1 tetramer virus or with SOAT1 dimeric virus expressed SOAT activities at levels even much higher than those found in the stable transfectant of HEK293 cells, demonstrating that the recombinant BacMam viruses infected 293-F cells is an excellent method for producing large amount of catalytically active recombinant SOAT1 (Supplementary Fig. 1b).

**Fig. 1:**
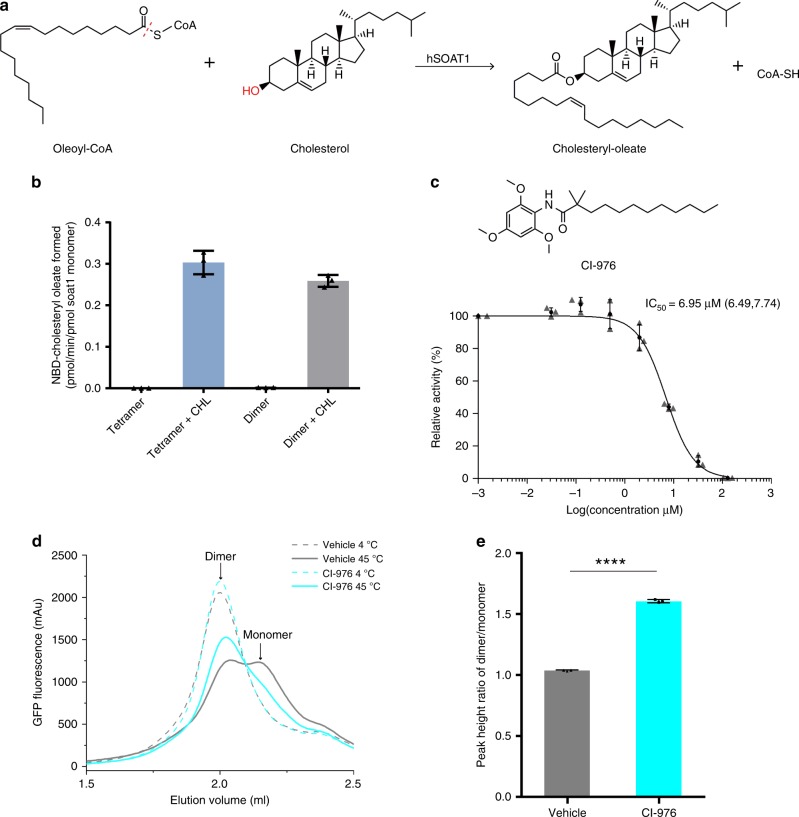
The enzymatic reaction catalyzed by hSOAT1. **a** Chemical structures of the substrates and products of hSOAT1 enzyme are shown. The red dashed line indicates the bond that is broken during acyl-transfer reaction. The hydroxyl group that accepts acyl group is highlighted in red. **b** The activation effect of cholesterol (CHL) on the esterification reaction of NBD-cholesterol catalyzed by hSOAT1 tetramer and dimer (Data are shown as means ± standard deviations, *n* = 3 biologically independent samples). **c** Chemical structure of CI-976 and dose-dependent inhibition curve of hSOAT1 tetramer by CI-976 (The first data point is an artificial point. Data are shown as means ± standard deviations, *n* = 3 biologically independent samples, and numbers in parentheses are the range for IC50 obtained from curve fitting). **d** Representative fluorescence-detection size-exclusion chromatography (FSEC) traces of N terminal GFP-tagged SOAT1 dimer at 4 °C (dashed lines) or 45 °C (solid lines) in the presence of 100 µM CI-976 (cyan) or DMSO alone (vehicle, gray). The elution positions of SOAT1 dimer and putative monomer are labeled by arrows. **e** The peak height ratio of the dimer/monomer in the presence of DMSO alone (vehicle, gray) or 100 µM CI-976 (cyan) (Data are shown as means± standard deviations, *n* = 3 biologically independent samples, ****p < 0.0001; two-tailed paired *t*-tests). Source data are provided as a Source Data file.

In order to measure the cholesterol-activated *O*-acyltransferase activity of the purified proteins, we developed the in vitro hSOAT1 assay using fluorescence-labeled NBD-cholesterol and oleoyl-CoA as substrates based on the previously reported SOAT1 whole cell assay^25^ (Fig. 1a and Supplementary Fig. 1c–h). The results show that the activity of hSOAT1 tetramer is linear within the first 15 min (Supplementary Fig. 1h), and both the purified tetrameric and dimeric hSOAT1 proteins showed cholesterol-activated *O*-acyltransferase activity in detergent micelles (Fig. 1b, Supplementary Fig. 1d–h). SOATs are allosteric enzymes that can be activated by cholesterol^4^ and it is predicted that SOATs have two functional distinct cholesterol binding sites. One site is the substrate-binding site and the other is the allosteric activating site that provides the feedback regulation mechanism regarding cholesterol concentration in the ER^4^. Our results suggest the NBD-cholesterol can act as a substrate while not the activator (Fig. 1b). Therefore, this assay provides the opportunities to study cholesterol activation effect. As expected, the hSOAT1 enzyme activity is inhibited by CI-976 in a dose-dependent manner (Fig. 1c). Moreover, we measured the thermostability of hSOAT1 dimer by fluorescence-detection size-exclusion chromatography (FSEC)^26^ (Fig. 1d) and found the solubilized hSOAT1 dimer dissociated into putative monomer upon heat treatment while the presence of CI-976 significantly enhanced the stability of hSOAT1 dimer (Fig. 1e).

**Fig. 2:**
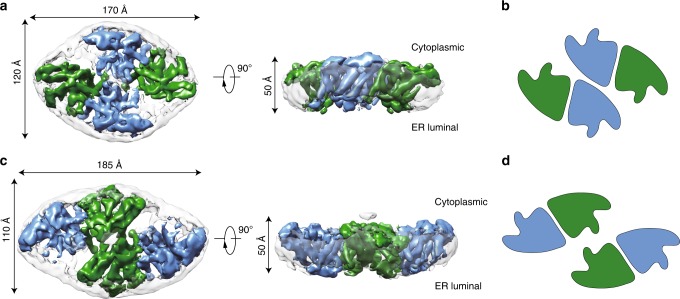
Cryo-EM maps of the human SOAT1 tetramer. **a** Cryo-EM density map of the oval-shaped hSOAT1 tetramer in top view and side view. Two subunits in one hSOAT1 dimer are colored in green and blue, respectively. Densities of the MSP and lipids in nanodiscs are colored in gray with semi-transparency. **b** The domain arrangement of oval-shaped hSOAT1 tetramer is shown in a cartoon model in top view. Each subunit is colored the same as in **a**. **c** The density map of the rhombic-shaped hSOAT1 tetramer is shown in top and side view. **d** The domain arrangement of rhombic-shaped hSOAT1 tetramer in top view. Each subunit is colored in the same way as in **c**.

**Fig. 3:**
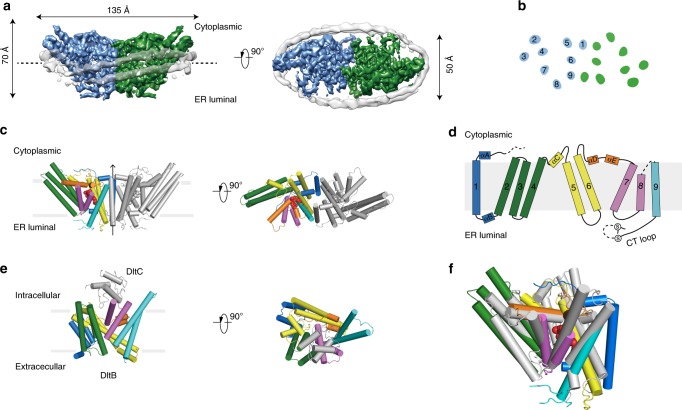
The structure of hSOAT1 dimer. **a** Cryo-EM map of hSOAT1 dimer in side view and top view. Two subunits of the dimer are colored in green and blue. Density corresponding to the nanodisc is colored in gray with semi-transparency. **b** Top view of the cross-section of the transmembrane domain at the approximate level indicated by the dashed lines in **a**. The identities of the transmembrane helices from the blue subunit are labeled in numbers. **c** The structural models of hSOAT1 dimer are shown in side view and top view. Helices are shown as cylinders. One subunit of hSOAT1 is in rainbow color and the other subunit is in gray. CI-976 molecule is shown as red spheres. **d** The topology of one hSOAT1 subunit. The colors are used in the same way as in **c**. **e** The crystal structure of DltB-DltC complex in side view and top view (PDB ID: 6BUG). The DltB subunit is in rainbow color while the DltC subunit is in gray. **f** The superposition of DltB (gray) onto one hSOAT1 protomer (colored).

To investigate the structure of hSOAT1, we prepared the sample of hSOAT1 in the presence of CI-976 in detergent micelles for cryo-EM analysis. The 3D classification showed the hSOAT1 sample had severe conformational heterogeneity (Supplementary Fig. 2). One of the 3D classes can be further refined to 12 Å and the map allow the visualization of the general shape of hSOAT1, in which the cytosolic N terminal oligomerization domain lays above the transmembrane domain. Moreover, the central slice of the transmembrane domain density map indicated the hSOAT1 is a tetramer composed of dimer of dimer (Supplementary Fig. 2d), which is consistent with previous biochemical data^21^. To further stabilize the structure of transmembrane domain, we reconstituted hSOAT1 into a lipid nanodisc (Supplementary Fig. 3a and b) for cryo-EM analysis (Supplementary Fig. 4). The top view of 2D class averages of the nanodisc sample had markedly enhanced features and confirmed the transmembrane region of hSOAT1 in nanodisc to be a dimer of dimer (Supplementary Fig. 4b). However, some 2D class averages in top views showed that one distinct dimer was adjacent to another blurry but still distinguishable dimer (Supplementary Fig. 4b), suggesting the dimer-dimer interface is mobile. Through multiple rounds of 2D and 3D classification, two 3D classes with discernable transmembrane helix densities were isolated. Subsequent refinements generated reconstructions at resolutions of 8.2 Å (for the oval-shaped tetramer) and 7.6 Å (for the rhombic-shaped tetramer) (Supplementary Fig. 4c, d). The oval-shaped structure occupies a 3D space of 170 Å×120 Å×50 Å (Fig. 2) and the shape is similar to 3D Class 1 observed in detergent micelles (Supplementary Fig. 2), and the rhombic-shaped structure occupies 185 Å×110 Å×50 Å (Fig. 2), similar to the 3D Class 2 observed in detergent micelles (Supplementary Fig. 2). The N terminal tetramerization domain is invisible in both maps probably due to its flexibility. These two structures reveal that the dimer-dimer interfaces of oval-shaped and rhombic-shaped hSOAT1 are distinct (Fig. 2b, d), correlating with the 3D heterogeneity observed in detergent micelles. This suggests the interfaces between dimers are unstable and dynamic in nature, which in turn hampered high-resolution structure determination. To overcome the conformational heterogeneity in the tetrameric hSOAT1 sample, the functional dimer construct (hSOAT1 66–550) was expressed and purified. The protein was reconstituted into the nanodisc in the presence of inhibitor CI-976 (Supplementary Fig. 3c, d). Subsequent 3D reconstruction generated a 3.5 Å cryo-EM map, which allowed the model to be built de novo (Fig. 3a, Supplementary Figs. 5 and 6, and Supplementary Table 1).

### Structure of hSOAT1 dimer

The hSOAT1 dimer has a symmetric rubber raft shape and occupies a 135 Å×70 Å×50 Å three-dimensional space (Fig. 3a). Each hSOAT1 subunit is composed of nine transmembrane helices (Fig. 3b), which is consistent with previous prediction based on the biochemical data^27^. The first 52 amino acids (66–117) of the dimeric hSOAT1 were invisible in the cryo-EM map, presumably due to their high flexibility (Fig. 3c, d). Notably, this region has the low-sequence conservation between SOATs from different species and paralogues. The cytosolic pre-αA loop runs parallel to the membrane. The amphipathic αA floats on the putative lipid bilayer with hydrophobic di-leucine motifs facing the membrane and connects to M1 via a near 90° turn (Fig. 3d). M1 is linked to the long tilted M2-M4 tri-helix bundle by an ER-luminal loop and a short helix αB (Fig. 3d). The six transmembrane helices M4-9 form a funnel-shaped central cavity, which is capped by the cytosolic helices αC, αD, and αE on the top. At the end of M9, a C-terminal ER luminal loop (CT loop) is cross-linked by the disulfide bond formed between C528 and C546 (Fig. 3d). It was previously reported that this loop is important for hSOAT1 activity and stability^28,29^. Indeed, the CT loop interacts with both the luminal M7-M8 loop and the M5-M6 loop (Fig. 3d) at the ER luminal side.

The TM2-9 of hSOAT1 share a similar structural fold with H4-H16 of DltB^18^, with core RMSD of 3.2 Å for 227 structurally aligned residues, despite of their low-sequence identity (Fig. 3e, f), suggesting a common evolutionary origin of the MBOAT family enzymes.

### Dimer interface

**Fig. 4:**
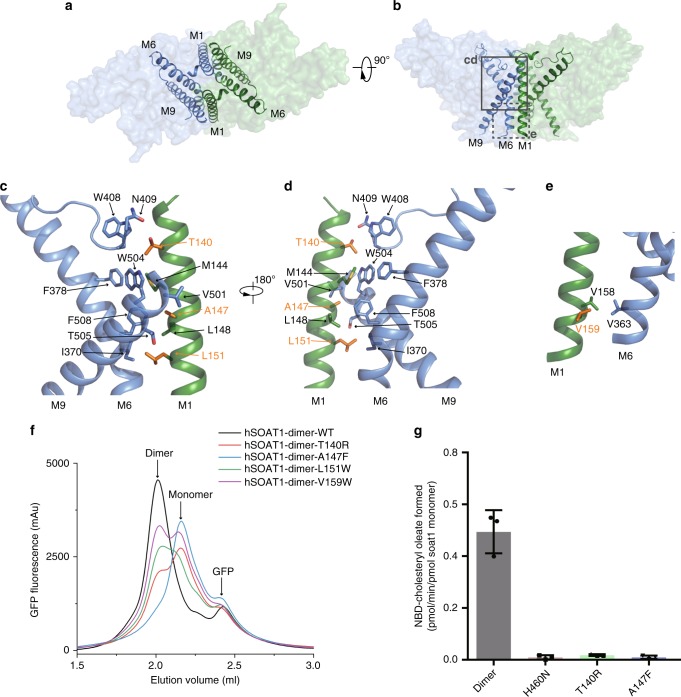
The dimer interface of hSOAT1 dimer. **a, b** Top view and side view of one hSOAT1 dimer are shown in surface representation with semi-transparency. M1, M6 and M9 helices are shown as ribbons. **c** Close-up view of the dimer interface boxed by solid lines in **b**. Interacting residues were shown in sticks. Residues mutated in **f** were shown in orange. **d** A 180° rotated view compared to **c**. **e** Close-up view of the interacting residues boxed by dashed lines in **b**. **f** FSEC traces of hSOAT1 dimer with interface mutations. **g** The activities of hSOAT1 dimer mutants measured using NBD-cholesterol as substrate, in the presence of cholesterol. Source data are provided as a Source Data file (Data are shown as means ± standard deviations, *n* = 3 biologically independent samples).

In contrast to the monomeric DltB protein^18^, the functional building block of hSOAT1 is a dimer, with a packing interface area of 6,520 Å^2^. The two hSOAT1 protomers interact through the M1, M6, M6-αD loop and M9 helices in a symmetric way (Fig. 4a, b). In the inner leaflet of the ER membrane, M144, A147, L148 and L151 on M1 of one subunit interact with I370 and F378 on M6, and V501, W504 and F508 of M9 of the other subunit (Fig. 4c, d). In the outer leaflet of ER membrane, V158 and V159 on M1 interact with V363 on M6 of the other subunit (Fig. 4e). The residues that form the dimer interface are mostly hydrophobic and interact with each other in a shape-complementary manner. To interrogate the function of the dimer in hSOAT1, we mutated several residues on the dimer interface to larger residues, including T140R, A147F, L151W and V159W, to disrupt dimer formation. FSEC results showed theses mutants have reduced dimers fractions while increased monomer fractions to different extent, with A147F and T140R having the most pronounced effect (Fig. 4f). We purified T140R and A147F mutants in hSOAT1 dimer construct and found they only exhibit little residual enzyme activities, at levels similar to that of the catalytic dead H460N mutant (Fig. 4g and Supplementary Fig. 1i). These results indicate the local dimeric architecture might be important for SOAT1 activity, correlating with the observation that disruption of dimer by Triton X-100 or octyl glucoside was associated with loss of activity^30^.

### Reaction chamber of hSOAT1

**Fig. 5:**
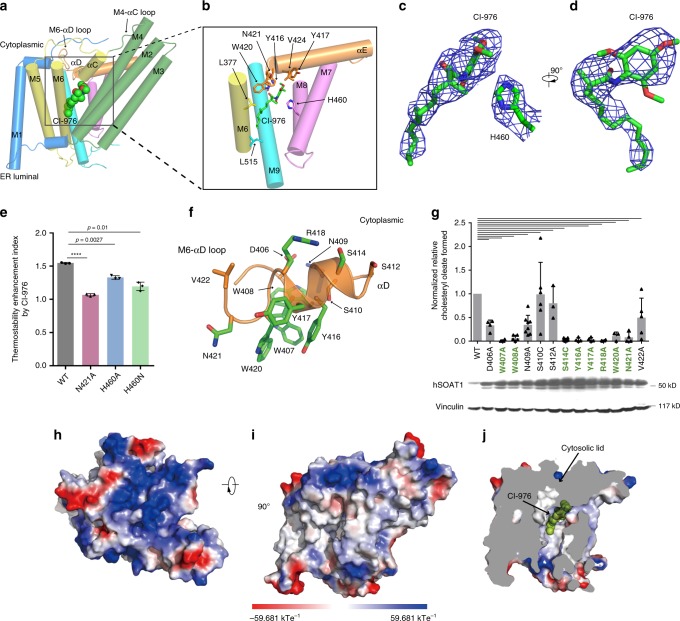
The catalytic chamber and the CI-976 binding site in hSOAT1. **a** Side views of one hSOAT1 subunit in cartoon representation. The transmembrane helices are colored in the same way as in Fig. 3c, d. The inhibitor CI-976 is shown in lemon sphere. **b** Close-up view of CI-976 binding sites. Residues that interact with CI-976 are shown as sticks. **c** Electron density of CI-976 and catalytic H460 were shown in blue meshes at the same contour level. Maps were further sharpened at −50 Å2 by Coot for visualization. **d** A 90° rotated view of c, density of H460 was omitted. **e** The thermostability enhancement effect of CI-976 on various hSOAT1 dimer mutants. The thermostability enhancement index was calculated by dividing dimer/monomer peak height ratio in the presence of CI-976 with the peak height ratio in the presence of DMSO alone (Data are shown as means± standard deviations, *n* = 3 biologically independent samples, ****p < 0.0001 for N421A; *p* = 0.0027 for H460A; *p* = 0.0100 for H460N respectively, two-tailed paired *t*-tests). **f** Close-up view of the M6-αD loop and αD with side chains shown in sticks. Residues that are important for SOAT1 enzyme activity reported in **g** were colored in green. **g** Enzymatic activities of various hSOAT1 mutants. (For D406A, *n* = 4. For W407A, *n* = 4. For W408A, *n* = 5. For N409A, *n* = 8. For S410C, *n* = 6. For S412A, *n* = 3. For S414C, *n* = 6. For Y416A, *n* = 4. For Y417A, *n* = 4. For R418A, *n* = 4. For W420A, *n* = 3. For N421A, *n* = 4. For V422A, *n* = 5 biologically independent samples.) Data are shown as means ± standard errors. A two-tailed unpaired t test was used to calculate the *p* values. For S410C, *p* = 0.9434. For S412A, *p* = 0.017. Other mutants had p values less than 0.0001. The expression levels of hSOAT1 mutants and endogenous vinculin are shown below. The original blots were provided as a Source Data file. **h**, **i** The top and side views of one hSOAT1 monomer in the surface representation. The surfaces are colored by electrostatic potential calculated by Pymol. **j** The cut-away view showing the binding pocket of the inhibitor CI-976 inside the reaction chamber. Source data are provided as a Source Data file.

Many acyltransferases utilize histidine as the catalytic base^31^. Previous studies suggest that the conserved H460 on M7 is crucial for hSOAT1 activity and is the putative catalytic residue^27^. Our structures show that the side chain of H460 points towards the interior of a central cavity cradled by M4−M9 (Fig. 5), suggesting this central cavity is the chamber where the acyl-transfer reaction takes place. In accordance with this, previous studies have also identified several residues in the central cavity that are important for hSOAT1 function. Mutations of residues on M7 and M8 affect the catalytic activity^32,33^. C467 at the end of M7 is the major target site for p-chloromercuribenzene sulfonic acid-mediated SOAT1 inactivation^34^. Intriguingly, most residues aligning the interior of reaction chamber are highly conserved (Supplementary Fig. 7b). These data also indicate that the local environment in the central cavity is important for the catalytic reaction.

The reaction chamber is covered by a lid formed by M4-αC loop, αC, M6-αD loop, αD and αE on the cytosolic side (Fig. 5a). Previous studies suggested that part of the cytosolic lid, residues 403–409 on M6-αD loop, may be involved in the binding of fatty acyl-CoA^35^. To further explore the role of the residues in this region, we performed alanine or cysteine mutagenesis of conserved residues within this region (amino acids 406–422 from M6-αD loop to αD, Fig. 5f and (Supplementary Fig. 7), and analyzed the effects of mutations after transient expressions of each of these mutant plasmid DNAs in a CHO cell clone AC29, that is devoid of endogenous SOAT activity, but regains enzyme activity upon transient expression of hSOAT plasmid DNA^36^. The results showed that mutating any of the following residues W407, S414, Y416, Y417, R418, W420, and N421 to alanines or cysteines caused loss in hSOAT1 enzyme activity (by greater than 90%) without severely lowering the cellular hSOAT1 protein expression in transfected cells (Fig. 5g). Because the hydrophilic side chains of S414 and R418 on αD face the cytosolic side of SOAT1, S414 and R418 might play important roles for hSOAT1 activity, such as binding of highly hydrophilic CoA group of fatty acyl-CoA substrate. These results further emphasize the important role of the cytosolic lid of the reaction chamber in the enzymatic reaction.

### Inhibitor and ligands binding sites

A strong extra non-protein density was found in the central cavity and the size and shape of the density matched that of the inhibitor CI-976, which was included during cryo-EM sample preparation (Supplementary Fig. 8a). By comparing the current map with the maps without CI-976 (as described later) (Supplementary Fig. 8), we proposed that this non-protein material represents the CI-976 molecule (Fig. 5a–d). The large trimethoxyphenyl head of CI-976 is sandwiched between the catalytic residue H460 on M7, and residues N421 as well as W420 on the αD-αE loop (Fig. 5b), all of which are crucial for the catalytic activity of hSOAT1. The head of CI-976 also interact with Y416 and Y417 on αD (Fig. 5b). The elongated dodecanamide tail of CI-976 extends in the cavity and interacts with L377 on M6 and L515 on M9 (Fig. 5b). In accordance with our assignment of CI-976, mutations of CI-976 interaction residues, N421A, H460A and H460N greatly diminished the enhancement effect of CI-976 on the thermostability of hSOAT1 dimer (Fig. 5e and Supplementary Fig. 9). The binding position of CI-976, right in the catalytic center, suggests that it inhibits the enzyme by precluding the loading of substrate into the catalytic center. This is consistent with the competitive behavior of CI-976 (ref.^15)^. Moreover, it has been reported that certain residues on M9 are responsible for the selectivity of subtype-specific SOAT inhibitors, such as pyripyropene A^37^. This further suggests that this large catalytic chamber can accommodate inhibitors with diverse chemical structures.

There are several extra non-protein densities observed in the cryo-EM map. One density (density A) is close to the dimer interface. It is surrounded by L129, L132 and L133 on the hydrophobic side of αA, F145 on M1, C333 on M5, F382 on M6 and W408 on M6-αD loop (Supplementary Fig. 8c). The shape of this density is similar to cholesterol, suggesting this ligand might be a tightly-bound sterol-like endogenous molecule or the head group of the digitonin detergent that was carried on during membrane protein extraction and purification. Previous work showed that SOAT1 exhibits only low affinity binding towards cholesterol, either as substrate or as activator, with dissociation constant at sub-millimolar concentration^23^. Therefore, we speculate that the molecule in density A may be neither a cholesterol substrate nor a cholesterol activator, but a sterol-like molecule, such as the head group of digitonin, bound to the enzyme in the sample preparation procedure. Another elongated density (density B) is inside the central cavity and surrounded by F258 and R262 on M4, F384 and W388 on M6, P304 on αC-M5 linker and V424 on αE (Supplementary Fig. 8e). One additional density (density C) is on the ER luminal side of the central cavity and surrounded by Y176 on αB, S519, W522 and Y523 on M9, L468 on M7-M8 linker, P250 on M3−M4 linker (Supplementary Fig. 8g). The exact identities of these densities A-C and their roles on the hSOAT1 function remain elusive (Supplementary Fig. 8i).

In order to gain more mechanistic insights into the catalytic mechanism of hSOAT1 and to trap the catalytic reaction in a transition state, we designed and synthesized a compound that might mimics the catalytic transition intermediate. Inspired by the previous work on GOAT^38^, we hypothesized that the catalytic reaction intermediate of hSOAT1 might be a ternary complex of sterol, acyl-CoA and the enzyme. Therefore, the covalent linkage of sterol and acyl-CoA would yield a competitive inhibitor with a higher affinity than each individual substrate alone. Pregnenolone was previously reported to be a substrate of hSOAT1 with a lower K_m_ and better solubility than cholesterol^39^. In the current study, CoA group was chemically covalently linked with stearoyl-pregnenolone to generate a bi-substrate analog for hSOAT1 (BiSAS) (Supplementary Fig. 10a). Indeed, BiSAS inhibited the purified hSOAT1 enzyme in the in vitro NBD-cholesterol based assay (Supplementary Fig. 10b). A cryo-EM sample of hSOAT1 dimer was prepared in the presence of BiSAS and cholesterol (Supplementary Fig. 3e, f) and the cryo-EM reconstruction generated a 3.5 Å map (Supplementary Figs. 11,12 and Supplementary Table 1). This map was compared with the CI-976 bound map and was found to be similar overall, with a real space correlation of 0.9. We anticipated that BiSAS might mimic both substrates of hSOAT1 and might occupy the substrate-binding pocket while cholesterol might only bind at the activating site. In contrast to our prediction, in the BiSAS map, the strong continuous density of CI-976 found in the central cavity of the CI-976 bound map was replaced by weak residual densities that were not continuous (Supplementary Fig. 8b), indicating the absence or low occupancy of BiSAS molecule, probably due to the low affinity or incompatibility of BiSAS in the nanodisc sample preparation conditions. In addition, we did not observe any extra cholesterol-like density that would suggest the presence of activating cholesterol either, probably due to the low affinity of activating cholesterol on hSOAT1 (ref.^23^). Instead, the BiSAS map most likely represents the apo resting state of hSOAT1. Interestingly, all three additional non-protein densities (density A−C) present in the CI-976 map were also observed in the BiSAS map (Supplementary Fig. 8d, f, h, j). Therefore, BiSAS map serves as a reference to validate the identity of CI-976 previously observed in the catalytic center and also suggests the tight associations of non-protein ligands A−C with the hSOAT1 protein and likely their functional importance as well.

## Discussion

SOAT catalyzes the esterification reaction between acyl-CoA and cholesterol. The surface representation of the hSOAT1 monomer shows an intra-membrane tunnel from outside of the molecule into the reaction chamber. The tunnel is located between M4 and M5, and is mainly hydrophobic. This lateral tunnel within the transmembrane domain of hSOAT1 might be the substrate or product transfer pathway for hydrophobic molecules. The other substrate, fatty acyl-CoA, is amphipathic with a hydrophobic tail and a highly hydrophilic CoA group. The cytosolic acyl-CoA can access the central reaction chamber only from the cytosolic side of hSOAT1. However, the surface representation of the hSOAT1 dimer shows that the reaction chamber is completely shielded from the cytosolic side by the two short αD-αE helices and associated intracellular loops (Fig. 5h–j). This suggests that the current structure represents a substrate-unbound resting state with relatively low catalytic activity, in which the putative catalytic residue H460 is less accessible to the acyl-CoA substrate (Supplementary Fig. 13). Therefore, acyl-CoA binding would induce a conformational change to open the reaction chamber and cholesterol binding at the allosteric activator site would induce a further conformational change for the sterol-dependent fully activation of hSOAT1 (ref.^4^) (Supplementary Fig. 13). Notably, a hSOAT1 tetramer structure in complex with oleoyl-CoA was solved by another group and reported on BioRxiv recently^40^ and there is indeed a conformational change of the reaction chamber to accommodate oleoyl-CoA substrate.

The structures of human SOAT1 presented here provide a high-resolution glimpse of the architecture and domain organization of this important enzyme and shed light on the structure of other closely related MBOAT family proteins, such as DGAT1. This work not only paves the way towards a better mechanistic understanding of SOAT1-catalyzed reaction, but also provides a template for structure-based inhibitor design to target several human diseases. Our current work shows that SOAT1 has 9 transmembrane domains (TMDs), with the active site His 460 located at the luminal half of M7. This is consistent with previous biochemical work^27^, and supports the idea that the cholesteryl esters produced by SOAT1 may first appear within the ER membrane before they become part of lipid droplets in the cytosol. Unexpectedly, though SOAT1 and SOAT2 shares extensive sequence homology, different groups reported that SOAT2 may contain only 2 or 5 TMDs, as reviewed in^41^. The uncertainty in the transmembrane topology of ACAT2 can be settled by further structural studies.

## Methods

### Cell culture

Sf9 insect cells (Thermo Fisher Scientific #12659017) were cultured in Sf-900 III serum-free medium (SFM; Thermo Fisher Scientific) or in SIM SF (Sino Biological) at 27 °C. HEK293F (Thermo Fisher Scientific #R79007) cells were cultured at 37 °C with 6% CO_2_ and 70% humidity in Free Style 293 medium (Thermo Fisher Scientific) supplemented with 1% fetal bovine serum (FBS). HEK293S cell line was kindly generated and provided by Professor Gobind Khorana at MIT^42^. The CHO mutant cell line AC29, deficient in ACAT1/SOAT1 activity, was isolated in Chang Lab^43^.

### Constructs

We cloned human SOAT1 (Hs_SOAT1), human SOAT2 (Hs_SOAT2), Xenopus laevis SOAT1 (Xl_SOAT1), chicken SOAT1 (Gg_SOAT1) and zebrafish SOAT1 (Dr_SOAT1) into NGFP tagged BacMaM vector for screening by FSEC method^44^ (Please see Supplementary Table 2 for primer list). The screening procedures identified human SOAT1 as a putative target with reasonable expression level and elution profile. cDNAs of human SOAT1 full-length (1–550), SOAT1-dimer (66–550) were cloned into a modified BacMaM vector, with N-terminal His_7_-strep-GFP tags^45^.

### SOAT1 activity assays using ^3^H-oleyl-CoA in vitro

The assay was conducted as described in^22^, by using solubilized cell extracts as the enzyme source and preformed taurocholate/cholesterol/phosphatidylcholine mixture and tritium-labeled oleoyl-CoA as the substrates. This assay provides large amounts of exogenous phospholipids and cholesterol at optimized concentrations, such that the membrane phospholipids and cholesterol endogenously associated with SOAT1 become significantly diluted and can no longer affect the enzyme activity in vitro. Cells were harvested by spinning down at 800 × *g* for 5 min at room temperature. After two washes with PBS, cell pellets were solubilized in 2.5% CHAPS, 1 M KCl in 50 mM KH_2_PO_4_ at pH 7.4 with protease inhibitors. 5 µl of cell homogenates at protein concentration of 2–5 mg/ml were diluted into 50 µl of preformed 9.3 mM taurocholate/1.6 mM cholesterol/11.2 mM phosphatidylcholine mixture and incubated on ice for 10 min. The reactions were initiated by adding 10 nmole of ^3^H-oleoyl-CoA. The reactions were continued at 37 °C for 10 min (ref.^23^).

### Thermostability assay

The hSOAT1 dimer mutants were generated by Quick Change methods (Please see Supplementary Table 2 for primer list). hSOAT1 dimer constructs were transfected into HEK293F cells (grown in FreeStyle 293 medium + 1% FBS, 37 °C) and the cells were harvested 48 h post transfection. The cells were washed with TBS buffer (20 mM Tris pH 8.0 at 4 °C, 150 mM NaCl) and incubated with 0.9% DMSO (vehicle group) or 0.9% DMSO together with 100 μM CI-976 (CI-976 group). Cells were solubilized by TBS with 1% GDN and protein inhibitors (2 µg ml^−1^ leupeptin, 2 µg ml^−1^ pepstatin A, 2 µg ml^−1^ aprotinin and 1 mM PMSF) at 4 °C for 30 min. Cell debris was removed by centrifugation at 13,000 × *g* for 10 min and centrifuged at 60,000 × *g* for 30 min. Each supernatant was split into two samples. One sample was kept at 4 °C as unheated sample and the other one was heated at 45 °C for 10 min using a thermo cycler. After heating, each sample was centrifuged at 60,000 × *g* for additional 30 min to remove precipitates and the supernatants were analyzed on a Superose 6 increase 5/150 (GE Healthcare) column running in 20 mM Tris pH 8.0 at 4 °C, 150 mM NaCl and 0.5 mM DDM. The fluorescence peak height at the hSOAT1 dimer elution position was divided by the peak height at the monomer elution position to generate the ratio which was used to quantify the thermostability. The enhancement effect of CI-976 on hSOAT1 dimer thermostability was quantified by dividing the ratio in the presence of CI-976 by the ratio in the presence of DMSO alone.

### Protein expression and purification

The BacMam expression system was used for large-scale expression of human SOAT1. The BacMaM viruses were added into suspended HEK293F cells (grown in FreeStyle 293 medium + 1% FBS, 37 °C). Sodium butyrate (10 mM) was added to the culture 12 h post-infection to promote protein expression and the temperature was lowered to 30 °C. Cells were harvested 60 h post-infection and washed with TBS buffer. Cell pellets were frozen at −80 °C for later use.

The cell pellets were resuspended in TBS buffer supplemented with protease inhibitors (2 µg ml^−1^ leupeptin, 2 µg ml^−1^ pepstatin A, 2 µg ml^−1^ aprotinin and 1 mM PMSF). Unless stated otherwise, all buffers used for purification were supplemented with inhibitor either 1 μM inhibitors CI-976 or BiSAS. The cells were broken by sonication and centrifuged at 5000 × *g* for 10 min with JA25.5 rotor (Beckman) to remove cell debris. The supernatant was centrifuged at 60,000 × *g* for 1 h in Ti45 rotor (Beckman) to harvest cell membrane in pellets. The membrane pellets were homogenized in TBS, solubilized by 1% digitonin for 2 h at 4 °C, and centrifuged at 60,000 × *g* for 1 h. The supernatant was loaded onto a 1 ml prepacked strep-tactin superflow high-capacity column (IBA) and washed using TBS buffer with 0.1% digitonin. The binding protein was eluted using TBS buffer with 0.1% digitonin and 10 mM desthiobiotin. The eluted proteins were digested with PreScission protease to remove tags and further purified by superose-6 increase column (GE Healthcare) in TBS buffer with 0.1% digitonin or 40 μM GDN. Buffers were not supplemented with inhibitors during the purification of proteins used for the activity assay. Purified protein were either directly used for experiments or flash frozen in liquid nitrogen for storage at −80 °C. Stored protein was thawed on ice and centrifuged to remove precipitates before further experiments. The original gels for protein purification were provided as a Source Data file.

### SOAT1 activity assays using ^3^H-oleate in intact cells

This assay is designed to measure the rate of ^3^H-cholesteryl oleate biosynthesis in intact cells (i.e., without having to break up the cells). Mutant CHO AC29 cells that lack endogenous SOAT activity were cultured in 6-well plates at 37 °C and transiently transfected with DNAs from various single amino acid substitutions of hSOAT1 as indicated. Unless stated otherwise, each construct contained the 6x His tag at the N-terminus. We used hSOAT1 that contained the C92A substitution as the wild type hSOAT1. Control experiments showed that the SOAT activity of the C92A mutant remained the same as the wild type^27^ but the C92A substitution greatly diminished the SOAT1 protein aggregation that occurred in vitro during the SDS-PAGE process. At the third day after transfections, the cells were split into three equal parts by trypsinization. One part was used to monitor the hSOAT protein expression by western blot analysis, using the SOAT1 specific antibodies DM10 as the probe, as described^46^, with the intensity of the WT hSOAT protein set as 1.0. The other two parts of the transfected cells were used to monitor the SOAT enzyme activity, by incubating the intact cells to 20 μl of ^3^H-oleate/fatty acid free BSA (7.5 × 10^6^ dpm μl^−1^) for 20 min. The amount of ^3^H-cholesteryl oleate produced was determined by the procedure previously described^27,36^. Briefly, after 3H-oleate/BSA pulse, cells were rinsed with PBS, harvested in 1 mL per well of 0.1 M NaOH, incubated at RT for 30 min. The cell homogenates were transferred to13 × 100 mm size glass tubes; then neutralized by adding 67 μl of 3 M HCl, and buffered with 50 μl of 1 M K_2_HPO_4_. 80 μg per tube of non-radiolabeled cholesteryl oleate was added as carrier for identification purposes. Cellular lipids in each tube were extracted with 3 mL of CHCl_3_:MeOH at 2:1 followed by adding 1 mL of H_2_O. The bottom chloroform phase that contained the lipid samples were dried under N_2_, then redissolved in 80 μl of ethyl acetate and spotted onto TLC plates (Anatech), with petroleum ether:ether:acetic acid (90:10:1) as the solvent system. After TLC, the plates were air-dried, the cholesteryl oleate bands (at Rf 0.9) were identified by briefly staining the TLC plates with iodine vapor. The cholesteryl oleate bands were scraped into scintillation vials and counted in a scintillation counter after addition of 3 mL per vial of Ecoscint O. The enzyme activity of each mutant hSOAT1 was estimated relative to that of WT hSOAT1, with the protein content of each mutant hSOAT1 normalized with that of the WT hSOAT1.

### SOAT1 activity assay using NBD-cholesterol

2.8 mM cholesterol/11.2 mM PC/18.6 mM taurocholate were mixed to generate the cholesterol donor^47^. The tetrameric or dimeric hSOAT1 enzyme was prepared in GDN detergent. First, 10 μl 2 M KCl, 5 μl 5% BSA, 1 μl SOAT1 protein (A_280_ = 0.5), 5 μl micelles, 40 μl TBS buffer with 40 μM GDN were mixed with TBS buffer with 0.5% CHAPS to reach the volume of 100 μl and incubated at 37 °C for 2 min. In order to measure the IC_50_ of inhibitors, different concentrations of given inhibitors were added, as indicated. Then, 1.0 μl 0.2 mg ml^−1^ NBD-cholesterol (Sigma, N2161) solubilized in 35% β-cyclodextrin (Sigma, HZB1102) was added and the mixture was incubated at 37 °C for 2 min. To start the enzymatic reaction, 1 μl 2.5 mM oleoyl-CoA (Sigma, O1012) was added and the reaction mixture was incubated at 37 °C for 15 min. The reaction was terminated by adding 2:1 chloroform/methanol, the extract was separated on an HPLC column at 0.2 ml/min (Agilent, Poroshell HPH-C18, 2.7 μm) running in 100% ethanol and detected via fluorescence detector on an HPLC (SHIMADZU). NBD-cholesterol eluted at 1.3 min and its ester eluted at 1.9 min. The peak areas of the NBD-cholesteryl ester products and remaining NBD-cholesterol were integrated separately to obtain the relative ratio of NBD-cholesterol that was converted into NBD-cholesteryl esters.

### Nanodisc preparation

MSP2X was constructed by linking two MSP1E3D1 by PCR overlap extension, with Gly-Thr as the linker. The MSP2X gene was constructed into a pET vector, with N-terminal His_6_ tag and HRV 3C site. The MSP2X and MSP2N2 proteins were purified by Talon resin^48^. The eluted SOAT1 protein in TBS buffer with 0.1% digitonin and 10 mM desthiobiotin from strep-tactin column was concentrated by a 100-kDa cut-off ultrafiltration device (Millipore) and exchanged into buffer without desthiobiotin. The SOAT1 protein was mixed with soybean polar lipids extract (SPLE, Avanti) and purified MSP (MSP2N2 for SOAT1 tetramer, MSP2X for SOAT1 dimer) at a molar ratio of SOAT1: MSP: SPLE = 1:7:100. For the hSOAT1 dimer in nanodisc that contained cholesterol, SPLE and cholesterol (at 4:1 ratio) were supplemented in GDN detergent micelles. The SOAT1 protein was mixed with micelles and purified MSP2X at a molar ratio of SOAT1: MSP2X: SPLE = 1:4:100. After incubating at 4 °C for 30 min, Bio-beads SM2 (Bio-Rad) were added and rotated at 4 °C for 1 h to initiate the reconstitution. Another batch of fresh bio-beads was added and rotated at 4 °C overnight. The next day, the Bio-beads were removed and the mixture was loaded into a streptactin column to remove the empty nanodisc. The eluted hSOAT1 in the nanodisc was concentrated and cleaved by Prescission protease to remove GFP tags. The nanodisc was centrifuged at 60,000 × *g* for 30 min, and then loaded onto a superose-6 increase column running in TBS buffer containing 0.5 mM TCEP. The collected fractions were detected by SDS−PAGE and peak fractions were concentrated to A_280_ = 1.2. Because the hSOAT1 tended to aggregate after nanodisc reconstitution, fractions of each peak were combined and concentrated for cryo-EM grids preparations and screening and only fractions containing high ratio of SOAT1 dimer were used for data collection.

### Cryo-EM data collection

The nanodisc samples were loaded onto glow-discharged GiG R1/1 holey carbon gold grids (Lantuo) and plunged into liquid ethane by Vitrobot Mark IV (Thermo Fisher Scientific). Cryo-grids were screened by Talos Arctica electron microscope (Thermo Fisher Scientific) operated at the voltage of 200 kV using a Ceta 16 M camera (Thermo Fisher Scientific). Optimal grids were transferred to Titan Krios electron microscope (Thermo Fisher Scientific) operated at the voltage of 300 kV, with an energy filter set to a slit width of 20 eV. Super-resolution movies (50 frames per movie) were collected with a dose rate of 5.4 e^−^pixel^−1^s^−1^ using K2 Summit direct electron camera (Thermo Fisher Scientific) at a nominal magnification of 130,000 ×, equivalently to a calibrated super-resolution pixel size of 0.5225 Å, and with defocus ranging from −1.3 μm to −2.3 μm. All data acquisition was performed automatically using SerialEM^49^.

### Cryo-EM image processing

For the CI-976 complex, super-resolution movie stacks were motion-corrected, dose-weighted and 2-fold binned by MotionCor2 1.1.0 using 9 × 9 patches^50^. Micrographs with ice or ethane contamination were manually removed. Contrast transfer function (CTF) parameters were estimated using Gctf v1.06 (ref.^51^). Particles were picked by Gautomatch (developed by Kai Zhang) and subjected to reference-free 2D classification. Unless otherwise stated, all classification and reconstruction were performed with Relion 2.0 (ref.^52^). Initial model was generated by cryoSPARC^53^ using the selected particles from 2D classification. The selected particles were further subjected to 3D classification using C1 symmetry. The particles selected from good 3D classes were re-centered and their local CTF parameters were determined using Gctf v1.06. These particles were further refined by cisTEM^54^ using C2 symmetry imposed. The resolution estimation was based on the Part.FSC curve in cisTEM at FSC = 0.143 cut-off. Local resolution estimation for hSOAT1 dimer and CI-976 complex was calculated using blocres^55^. For the apo state, images were processed in the same way, except the finial refinement were done using Relion 3.0 (ref.^56^) with a soft mask that excluded the MSP and lipids. The resolution estimations of the apo state were based on the gold standard FSC of 0.143 cut-off after correction of the masking effect^57^. Local resolution estimation for hSOAT1 dimer in the apo state was calculated using Resmap^58^.

### Model building and refinement

The sharpened map in the presence of CI-976 from cisTEM was converted into mtz file by Phenix^59^. The model was manually built de novo in Coot^60^. The assignments of transmembrane domain helices were based on their connectivity aided by the less-sharpened map. The register assignment and modeling building were based on the features of large aromatic side chains and partly aided by the further sharpened map. The manually built model was refined by Phenix^59^. The sterol-like ligand in non-protein density A was built as cholesterol for visualization. The model in the presence of CI-976 were fitted into the map in the presence of BiSAS by Chimera^61^ and further refined by Phenix.

### ConSurf calculation

The SOAT1 sequences (TF105767) were download from Treefam^62^, aligned by Clustal Omega and uploaded into the Consuf server^63^ together with the CI-976 pdb files. The figures were prepared by UCSF Chimera^61^.

### Chemical synthesis of BiSAS

To the solution of α-bromo stearic acid (727 mg, 2 mmol, 1.0 equiv), pregnenolone (632 mg, 2 mmol, 1.0 equiv) and dicyclohexylcarbodiimide (DCC, 495 mg, 2.4 mmol, 1.2 equiv) in dichloromethane (DCM, 30 mL) was added 4-dimethylaminopyridine (DMAP, 293 mg, 2.4 mmol, 1.2 equiv). The solution was stirred at room temperature for 24 h. The crude product was purified by column chromatography (Hexanes: Ethyl acetate = 10:1) to obtain the α-bromo ester (747 mg, 57 % yield) as a white solid. ^1^H NMR (400 MHz, CDCl_3_) *δ* 5.39 (d, *J* = 5.1 Hz, 1H), 4.67 (qd, *J* = 11.3, 9.5, 4.2 Hz, 1H), 4.17 (t, *J* = 7.4 Hz, 1H), 2.54 (t, *J* = 8.9 Hz, 1H), 2.40–2.32 (m, 2H), 2.12 (s, 4H), 2.08–1.84 (m, 6H), 1.75–1.58 (m, 4H), 1.56–1.39 (m, H), 1.37–1.11 (m, 32H), 1.03 (s, 3H), 0.88 (t, *J* = 6.7 Hz, 3H), 0.63 (s, 3H). ^13^C NMR (101 MHz, CDCl_3_) *δ* 209.69, 169.49, 139.39, 122.83, 77.48, 77.16, 76.84, 75.51, 63.81, 56.96, 49.99, 46.72, 44.12, 38.92, 37.69, 37.04, 36.74, 35.04, 32.08, 31.94, 31.91, 31.71, 29.84, 29.83, 29.81, 29.73, 29.61, 29.52, 29.45, 28.97, 27.61, 27.40, 24.62, 22.97, 22.84, 21.18, 19.46, 14.28, 13.37. HRMS(ESI): m/z calcd for C_39_H_66_BrO_3_^+^ [M + H]^+^: 661.418985, found 661.420600

To the solution of α-bromo ester obtained above (27 mg, 40 μmol, 1.0 equiv) and CoA-SH (62 mg, 80 μmol, 2.0 equiv) in *N,N*-dimethyllformamide (DMF, 1 mL) was added triethylamine (TEA, 56 μL, 0.4 mmol, 10 equiv). The solution was stirred under nitrogen atmosphere at 35 °C overnight. The crude product was purified by reverse phase HPLC (Water: Acetonitrile = 50: 50 to 5: 95) to obtain BiSAS in bis(triethylammonium) salt form (23.8 mg) as a colorless solid. ^1^H NMR (500 MHz, D_2_O) *δ* 8.44 (s, 1H), 8.06 (s, 1H), 6.04 (s, 1H), 5.27 (s, 1H), 4.89–3.72 (m, 9H), 3.65–3.20 (m, 6H), 3.10 (q, *J* = 7.2 Hz, 8H), 2.89–1.73 (m, 19H), 1.42–1.00 (m, 48H), 0.88 (s, 3H), 0.77 (s, 6H), 0.72–0.60 (m, 3H), 0.50 (s, 3H). HRMS(ESI): m/z calcd for C_60_H_99_N_7_O_19_P_3_S^−^ [M - H]^−^: 1346.593480, found 1346.591330.

### Quantification and statistical analysis

Global resolution estimations of cryo-EM density maps are based on the 0.143 Fourier Shell Correlation criterion^64^. Fluorescence values were plotted versus the log of the concentration of inhibitor, and GraphPad Prism 6 was used to generate a curve fit with dose-response inhibition equation: Y = 100/1 + 10^[Log(IC50−X) *HillSlope]^. IC_50_ values were calculated from the curve fit using GraphPad Prism software. The number of biological replicates (N) and the relevant statistical parameters for each experiment (such as mean or standard error) are described in the figure legends. No statistical methods were used to pre-determine sample sizes.

### Reporting summary

Further information on research design is available in the Nature Research Reporting Summary linked to this article.

## Supplementary information

Supplementary Information

Peer Review File

Reporting Summary

## Data Availability

Data supporting the findings of this manuscript are available from the corresponding author upon reasonable request. A reporting summary for this Article is available as a Supplementary Information file. The source data underlying Figs. 1b, c, e, 4g, 5e, g, and Supplementary 1b, f, h, 3b, d, f, 9e, 10b are provided as a Source Data file. The cryo-EM maps of hSOAT1 tetramer in oval shape, hSOAT1 tetramer in rhombic shape, hSOAT1 dimer bound with CI-976 and in the apo resting state have been deposited in the EMDB under ID codes EMD-0829, EMD-0830, EMD-0831 and EMD-0832. The atomic coordinates of hSOAT1 dimer bound with CI-976 and in the apo resting state have been deposited in the PDB under ID codes PDB 6L47 [10.2210/pdb6L47/pdb] and PDB 6L48 [10.2210/pdb6L48/pdb].

## Source Data

Source Data
