# Supplementary material for: Structural insights into the inhibition mechanism of human sterol O-acyltransferase 1 by a competitive inhibitor
Source: Nat Commun. 2020 May 18;11:2478. doi: 10.1038/s41467-020-16288-4 (PMC7234994; doi:10.1038/s41467-020-16288-4)
Supplement: Supplementary file 1 — Supplementary Information [file 41467_2020_16288_MOESM1_ESM.pdf]

Supplementary Information for

Structural insights into the inhibition mechanism of human sterol O-acyltransferase 1 by a competitive inhibitor

by Chengcheng Guan et al.

This PDF file contains:

Supplementary Figure 1-13

Supplementary Table 1-2

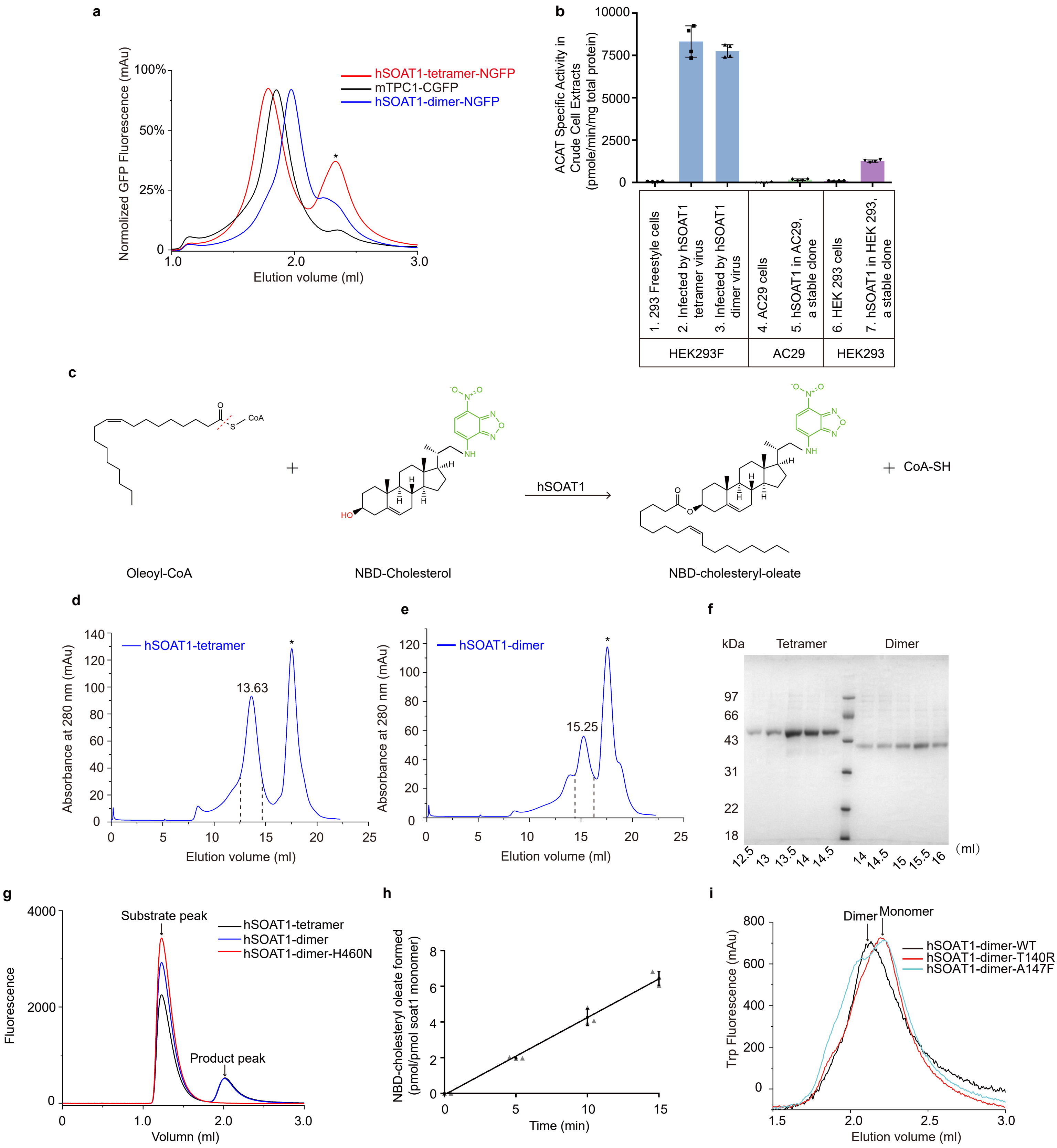

Supplementary Figure 1 | Characterization of human SOAT1 proteins. **a**, Fluorescence-detection size-exclusion chromatography (FSEC) traces of the N-terminal GFP tagged hSOAT1 tetramer and dimer on a Superose 6 increase column. The traces of C-terminal GFP tagged mouse TPC1 were shown in black. The NGFP-hSOAT1 tetramer protein elutes at a position slightly earlier than the dimeric mTPC1-CGFP, while the NGFP-hSOAT1 dimer protein elutes later than the mTPC1-CGFP. An asterisk denotes the position of free GFP. **b**, The activity assay of hSOAT1 tetramer and dimer in different crude cell extracts using tritium-labeled oleoyl-CoA as substrate. Data are shown as means ± SEM, n = 4 technical replicates. The experiments were repeated twice with similar results. **c**, The chemical reaction of hSOAT1 activity assay using NBD-cholesterol as substrate. The red dashed line indicates the bond that is broken during acyl-transfer reaction, the hydroxyl group that forms ester bond with the acyl group is highlighted in red. The NBD-fluorescent group is colored in green. **d-e**, The superose 6 elution profiles of hSOAT1 tetramer (**d**) and dimer (**e**), the fractions between the dashes were pooled and used for SDS-PAGE analysis. An asterisk denotes the position of GFP. **f**, The SDS-PAGE gel of purified hSOAT1 tetramer and dimer. The experiment has been repeated three times with success. **g**, The separation of NBD-cholesterol and NBD-cholesteryl-oleate by HPLC. Peak A is the free NBD-cholesterol. Peak B is the NBD-cholesteryl-oleate product. The fraction of NBD-cholesteryl-oleate product was calculated as area A/(area A+ area B). **h**, The reaction of hSOAT1 tetramer was linear with time within the first 15 min (Data are shown as means ± standard deviations, n = 3 biologically independent samples). **i**, FSEC traces of purified non-tag WT, T140R and A147F mutants of hSOAT1 dimer constructs.

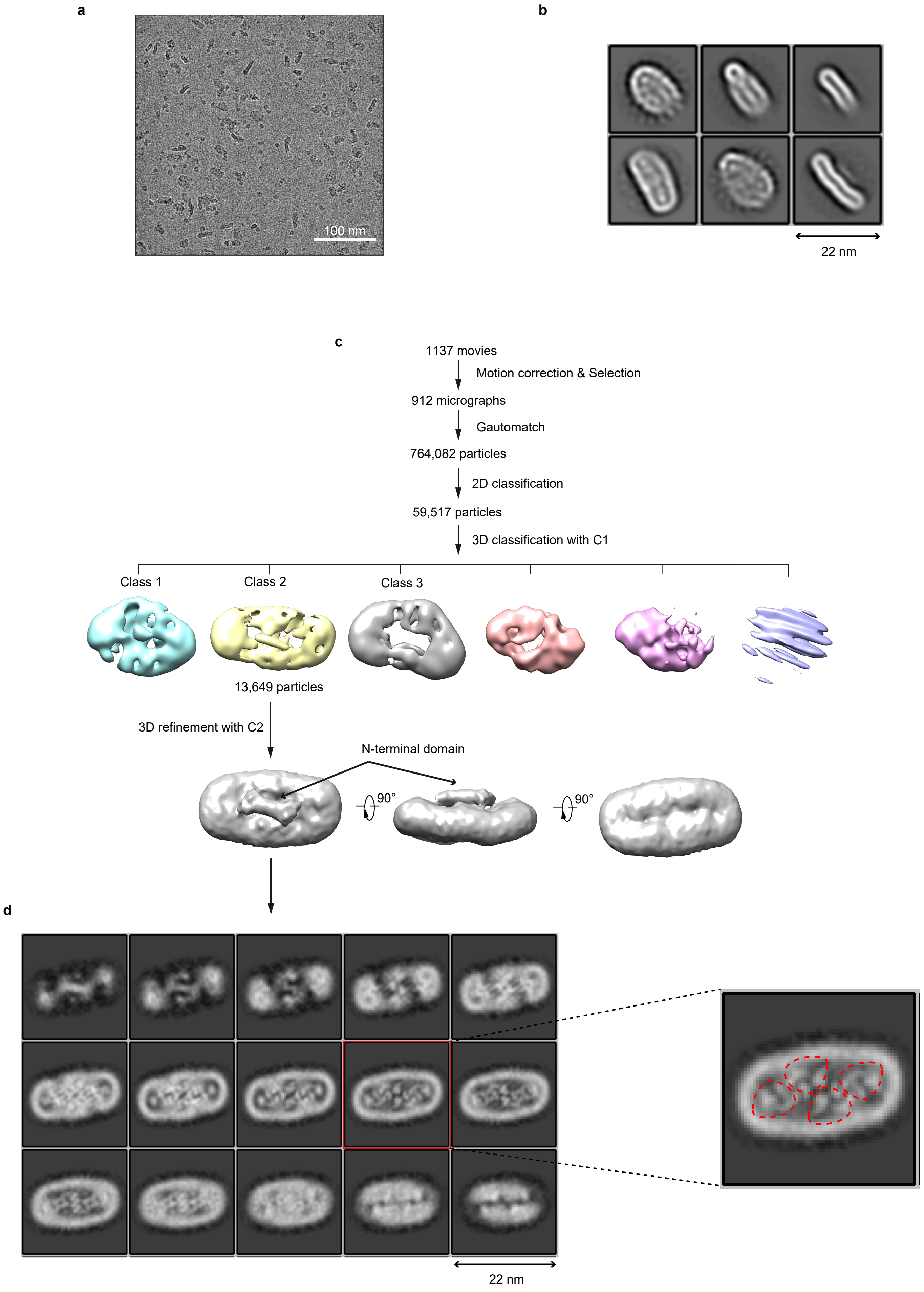

Supplementary Figure 2 | Cryo-EM image processing procedure of the hSOAT1 tetramer in digitonin detergent. a, One representative raw micrograph of hSOAT1 tetramer sample out of 1137 micrographs collected. **b**, Representative 2D class averages of the cryo-EM particles of hSOAT1 tetramer. **c**, Flowchart of the image processing procedure for hSOAT1 tetramer. **d**, The top-down slice view of the 3D density map after 3D refinement and postprocessing. The slice in red box is zoomed in for visualization. The red dashes circle each individual hSOAT1 monomer.

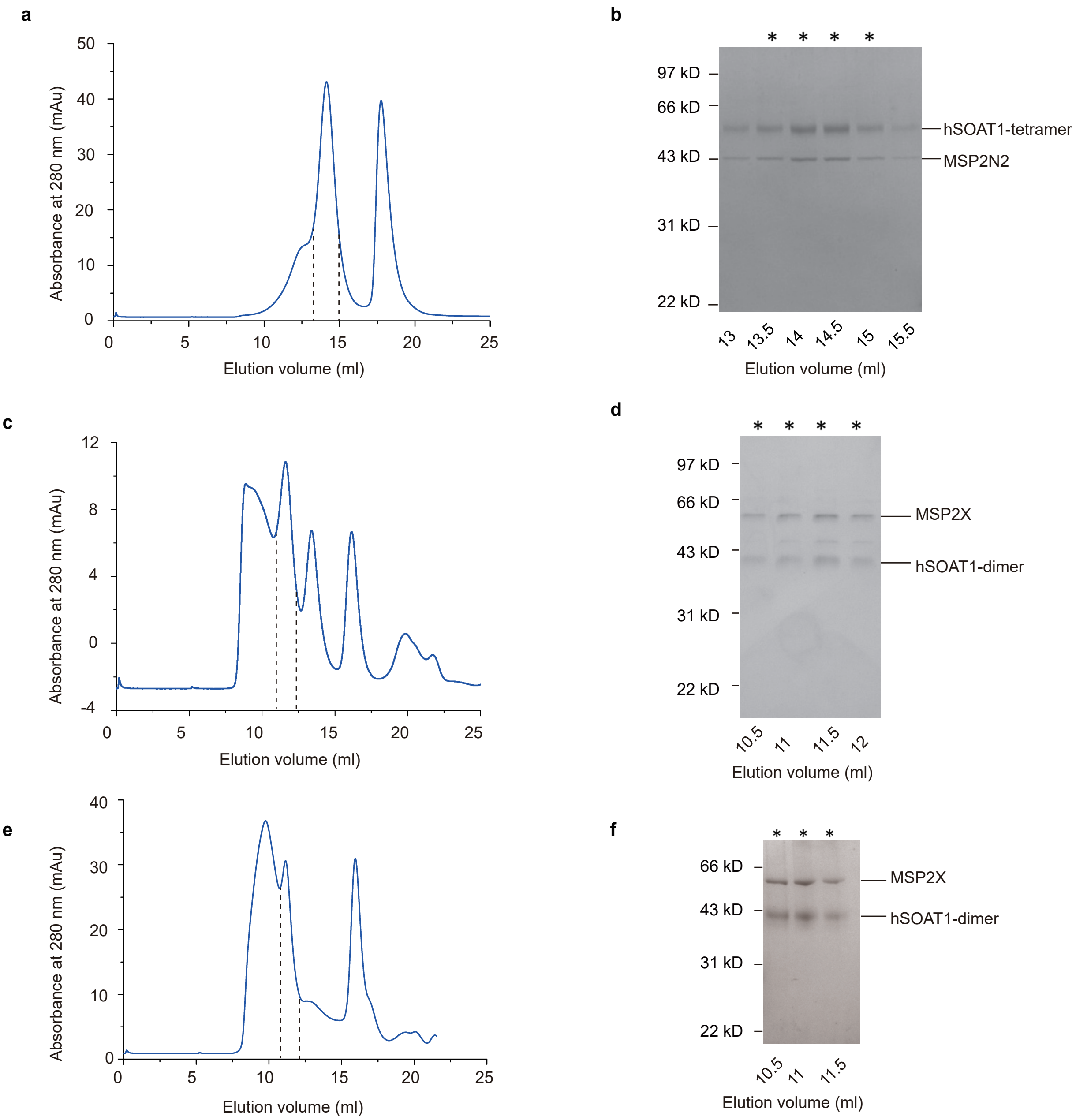

Supplementary Figure 3 | Purification of hSOAT1 tetramer and dimer nanodisc samples. a, Size exclusion chromatography (SEC) profile of the hSOAT1 tetramer nanodisc sample on Superose 6. The fractions between the dashes were pooled and used for cryo-EM analysis. **b**, hSOAT1 tetramer nanodisc samples of the indicated SEC fractions were subjected to SDS–PAGE and Coomassie blue staining. The asterisks denote the pooled fractions. The experiment has been repeated three times with success. **c-d**, Superdex 200 SEC profiles and SDS-PAGE of hSOAT1 dimer nanodisc in the presence of CI-976. The experiment has been repeated three times with success. **e-f**, SEC and SDS-PAGE results of hSOAT1 dimer nanodisc in the presence of cholesterol and BiSAS. The experiment has been repeated three times with success.

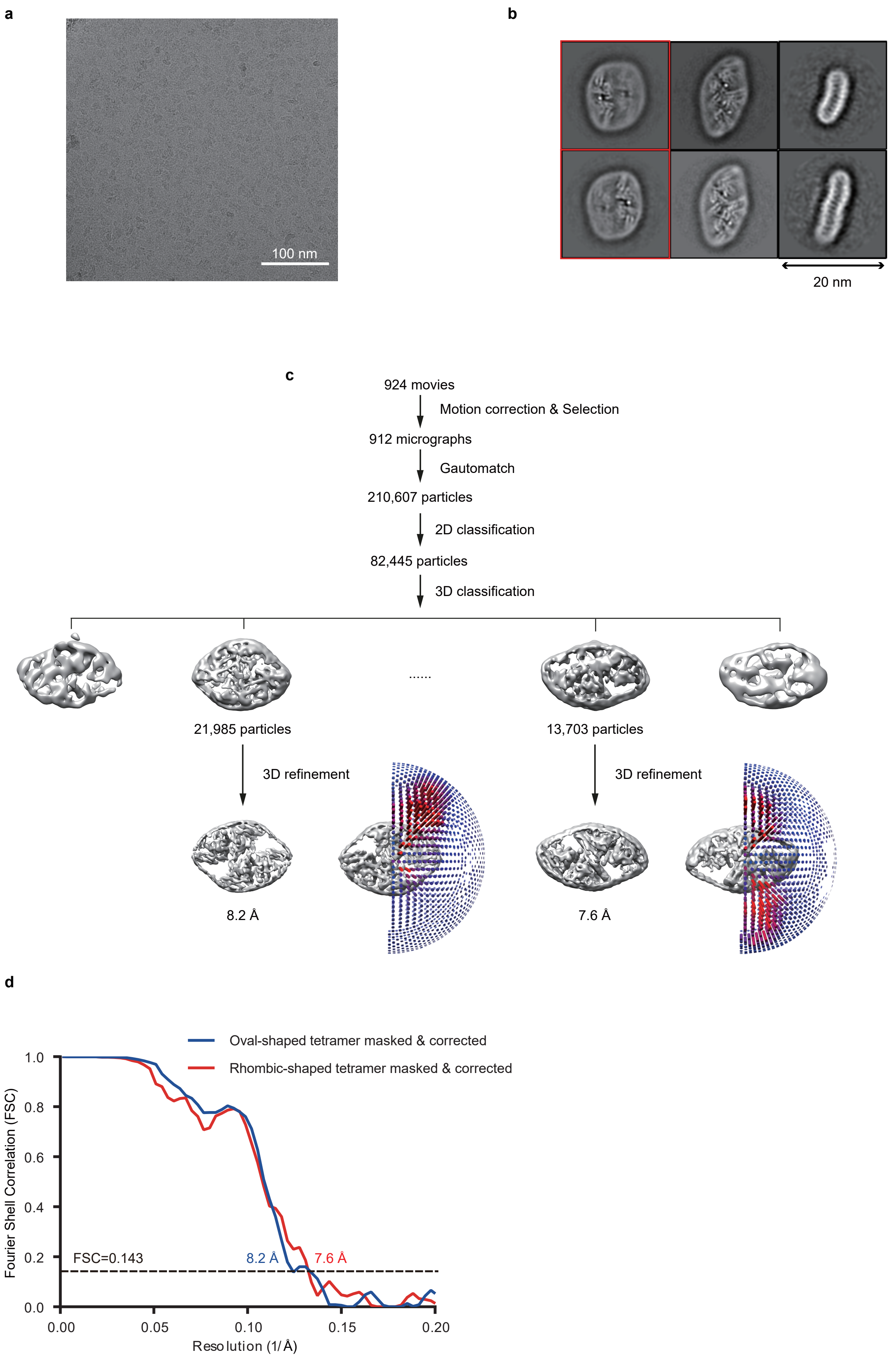

Supplementary Figure 4 | Cryo-EM image processing procedure of the hSOAT1 tetramer. **a**, One representative raw micrograph of hSOAT1 tetramer sample out of 924 micrographs collected. **b**, Representative 2D class averages of the cryo-EM particles of hSOAT1 tetramer. The 2D class averages in red boxes show one clear dimer in adjacent to a blurry dimer, indicating the highly mobile interface between dimers. **c**, Flowchart of the image processing procedure for hSOAT1 tetramer. **d**, Gold-standard Fourier shell correlation (FSC) curves of the final refined maps for oval-shaped tetramer (blue line) and rhombic-shaped tetramer (red line). Resolution estimations (8.2 Å for the oval-shaped tetramer and 7.6 Å for the rhombic-shaped tetramer) are based on the criterion of an FSC cutoff of 0.143.

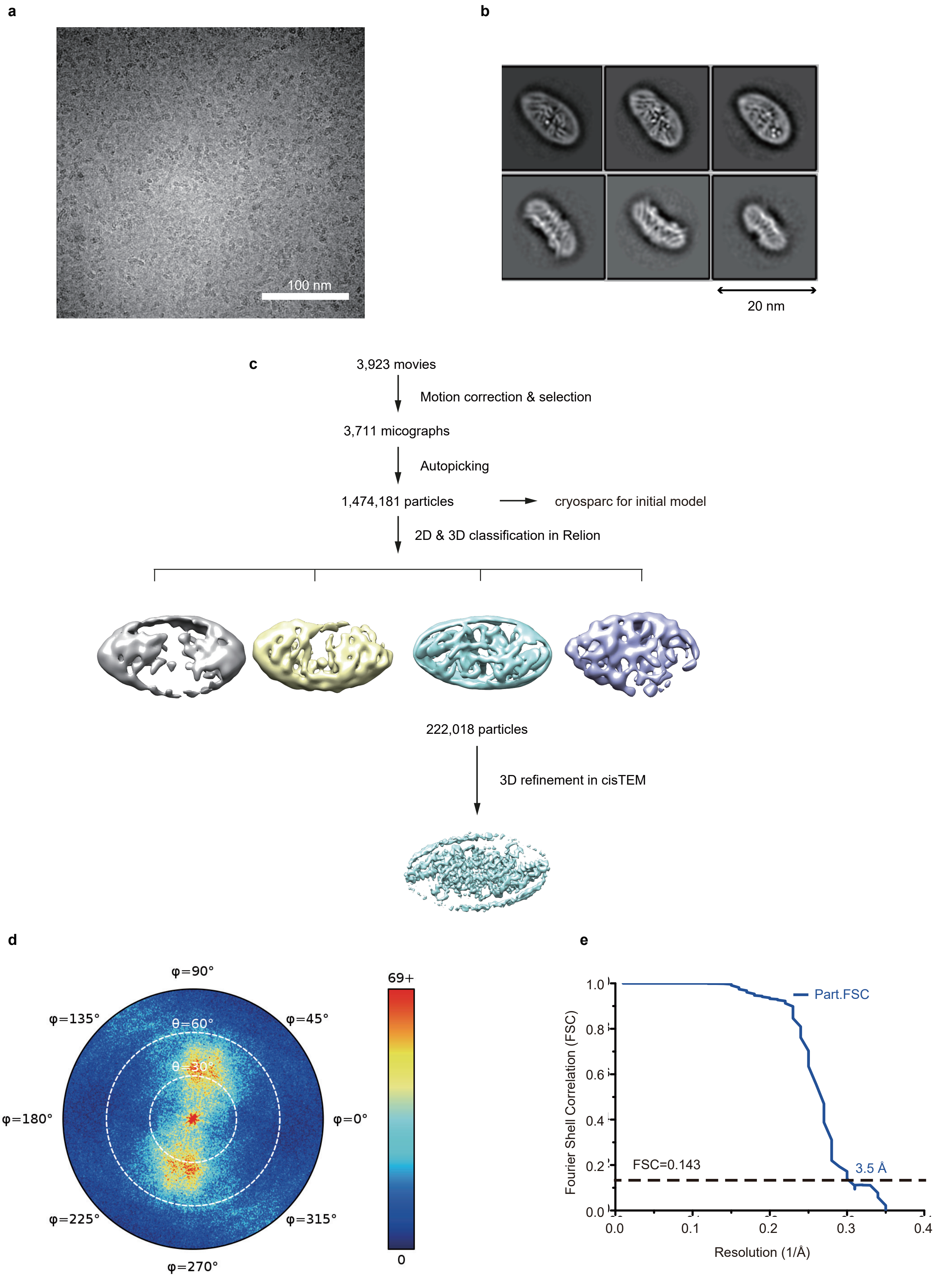

Supplementary Figure 5 | Cryo-EM image processing procedure of the hSOAT1 dimer in complex with CI-976. a, One representative raw micrograph of hSOAT1 dimer out of 3923 micrographs collected. **b**, Representative 2D class averages of the cryo-EM particles of hSOAT1 dimer. **c**, Flowchart of the image processing procedure for hSOAT1 dimer. **d**, Angular distribution of the final reconstruction of hSOAT1 dimer. **e**, Gold-standard Fourier shell correlation (FSC) curve of the final refined map for hSOAT1 dimer. Resolution estimation (3.5 Å) is based on the criterion of the FSC cutoff at 0.143 in cisTEM.

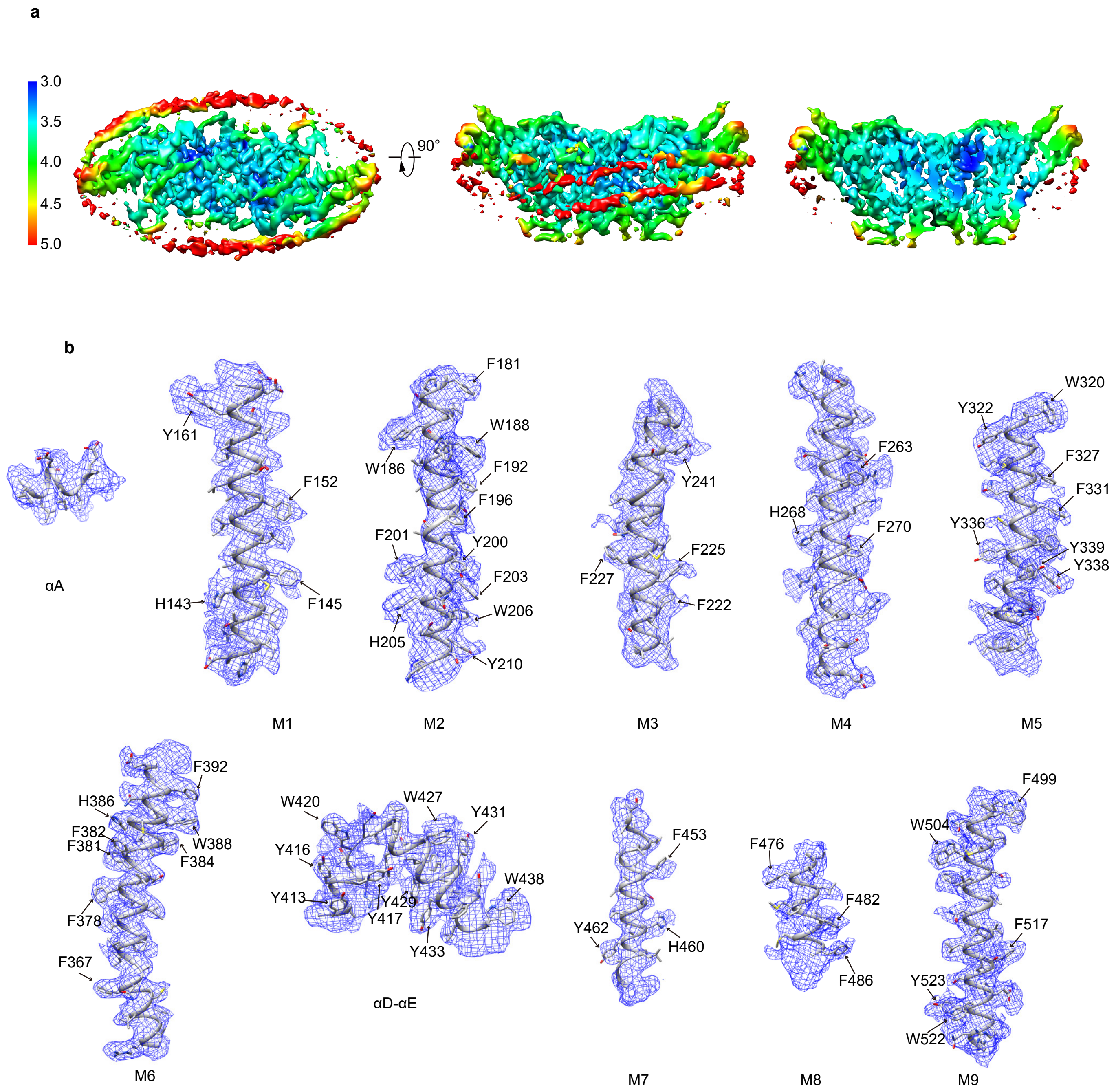

Supplementary Figure 6 | Electron density map of the hSOAT1 dimer in complex with CI-976. **a**, Top view (left), side view (middle) and cut-away (right) representations of the hSOAT1 dimer cryo-EM density map colored according to the local resolution estimation. **b**, EM density segments (blue mesh) of the 9 transmembrane helices (M1–M9), αA and αD-αE.

a

```
Hs_SOAT1  1   -------MVGEEKMSLRNRLSKSRE---NPEEDE-DQRNPAKESLETPSNGRIDIKQLIAKKIKLTAEAEELKPFFMKEVGSHFDDFVTNLIEKSASLDN 89
Hs_SOAT2  1   ----------MEPGGARLRLQRTEG----LGGER-ERQPCGDGNTET---------HRAPDLVQWTRHMEAVKAQLLEQAQGQLRELLDRAMREAIQSYP 76
Gg_SOAT1  1   MKALFVVAAMAGEDCVRKRPSGSTTTYKSPENEEMQRRPDGDRSFQNSSNGRVDVDHVITRKMQLIAEAEQLKPVFMKEVDSHFTEFVNSLVAKSALLDS 100
Xl_SOAT1  1   -------MSDEEGRVLRSRRLISKQSVHSEQEDV-ARRTEGDRVFRS--NGLVDLEHVINRKMEMRKKAEHLKNELMKEVDGHLNEFVNNLIEESTVLES 90
Dr_SOAT1  1   -------MVNEGAGGPRSRKSSHKV---ASSSDK-DGPERDAEEFPLSSNGKVEVEQIISAKLQLKKKAEHLKADLMRQFDAQVNEFMDSLIEESASLGS 89
Hs_DGAT1  1   ---------MGDRGSSRRRRTGSR-----PSSHG-GGGPAAAEE---------------------------------------------EVRDAAAGPD 39

                                                    αA                 M1                          αB
Hs_SOAT1  90  GGCALTTFSVLEGEKNNHRAKDLRAPPEQGKIFIARRSLLDELLEVD----HIRTIYHMFIALLILFILSTLVVDYIDEGRLVLEFSLLSYAFGKFPTVV 185
Hs_SOAT2  77  SQDK-PLPPPPPG----SLSRTQEPSLGKQKVFIIRKSLLDELMEVQ----HFRTIYHMFIAGLCVFIISTLAIDFIDEGRLLLEFDLLIFSFGQLPLAL 167
Gg_SOAT1  101 SSSA-SLFPASCSEKELHKAKALRAPPEHGKIFTARRSLLDELFEVS----HIRTIYHMFIALLIVFILSTLLVDFIDEGRLVLGFDLLVYVFGKFPVVF 195
Xl_SOAT1  91  MSAA---PSSGNVEKDSNKLRGLRAPPEHGKLFVSRRSLLDELFEVN----HIRTIYHMFIALLILFILSTLVVDCIDEGRLVLEFDLLVYAFGKFPIVI 183
Dr_SOAT1  90  SHVS-TVFPLSDKEK--SKLRNAQPSQGQSKQFVIRRSLLDDLFEVN----HIRTIYHMFIALLILFIFSTLVVDFIDEGRLVLEFDLLVYAFGQFPLVV 182
Hs_DGAT1  40  VGAAGDAPAPAPN-------KDGDAGVGSGHWELRCHRLQDSLFSSDSGFSNYRGILNWCVVMLILSNARLFLENLIKYGILVDPIQVVSL-FLKDPYS- 130

                         M2                                 M3                                  M4
Hs_SOAT1  186 WTWWIMFLST---FSVPYFLFQHWATGYSKSSHPLIRSLFHGFLFMIFQIGVLGFGPTYVVLAYTLPPASRFIIIFEQIRFVMKAHSF------VRENVP 276
Hs_SOAT2  168 VTWVPMFLST---LLAPYQALRLWARGTWTQATGL------GCALLAAHAVVLCALPVHVAVEHQLPPASRCVLVFEQVRFLMKSYSF------LREAVP 252
Gg_SOAT1  196 CTWLCMFCAT---VIIPYSLFSQWAQGYCSSSHRVIYSLFYGTLFTLFQTVGLGIGPTYVAISYALPPASRFIVILEQVRLVMKAHSF------VRENVP 286
Xl_SOAT1  184 STWLCMFLST---FIIPYGLFSTWARGYRASSHRSIRSIFFGILFMMFQTLGLGFMPTYIVLHHDLPPASRFIIILEQVRLTMKAHSF------VRENVP 274
Dr_SOAT1  183 VTWMCMFLST---LVVPYVLLVVWAGIYSSSQHRLLWTVLAGSILLIYQGLGLGFLPTYVVLKNGLPPASCFILILEQVRLMMKSHSF------IRENVP 273
Hs_DGAT1  131 WPAPCLVIAANVFAVAAFQVEKRLAVGALTEQAGLL-------LHVANLATILCFPAAVVLLVESITPVGSLLALMAHTILFLKLFSYRDVNSWCRRARA 223

                                 αC                                    M5
Hs_SOAT1  277 RVLNSAKEKSSTVPIPTVNQY---------LYFLFAPTLIYRDSYPRNPTVRWGYVAMKFAQVFGCFFYVYY---IFERLCAPLFRNIKQEPF---SARV 361
Hs_SOAT2  253 GTLRARRGEG--IQAPSFSSY---------LYFLFCPTLIYRETYPRTPYVRWNYVAKNFAQALGCVLYACF---ILGRLCVPVFANMSREPF---STRA 335
Gg_SOAT1  287 RVLSSVKEKSSSVPIPRISQY---------LYFLFAPTLIYRDNYPRNPMVRWGYVATKFAQVLGSLFYAYY---IFVRLCIPQFRNSSQETF---NLRG 371
Xl_SOAT1  275 RIFAFTKEKSSIVPVPQVTQY---------LYFLFAPTLIYRDNYPRNPSIRWGYVATKFAQVLGCLFYAYY---VFVRLCIPLFRNISQEPF---SLRV 359
Dr_SOAT1  274 RVRSLDRNKSNLVAVPQFTQY---------IYFLFAPTLIYKDNYPRNPCIRWGYVATKFSQVLGSLFYAYY---IFVRLCIPQFRNISMQLF---DPRA 358
Hs_DGAT1  224 KAASAGK-KASSAAAPHTVSYPDNLTYRDLYYFLFAPTLCYELNFPRSPRIRKRFL---LRRILEMLFFTQLQVGLIQQWMVPTIQN-SMKPFKDMDYSR 318

                           M6                                         αD          αE                 M7
Hs_SOAT1  362 LVLCVFNSILPGVLILFLTFFAFLHCWLNAFAEMLRFGDRMFYKDWWNSTSYSNYYRTWNVVVHDWLYYYAYKDFLWFFSKRFKSAAMLAVFAVSAVVHE 461
Hs_SOAT2  336 LVLSILHATLPGIFMLLLIFFAFLHCWLNAFAEMLRFGDRMFYRDWWNSTSFSNYYRTWNVVVHDWLYSYVYQDGLRLLGARARGVAMLGVFLVSAVAHE 435
Gg_SOAT1  372 LVLCIFNSILPGVLILFLVFFAFLHCWLNAFAEMMRFADRMFYKDWWNSTSYANYYRTWNVVVHDWLYYYAYRDFLWFFGKKFKAAAMLSVFTVSAAVHE 471
Xl_SOAT1  360 LVLCIFNSILPGVLVLFLAFFAFLHCWLNAFAEMLRFADRMFYKDWWNSTSFANYYRTWNVVVHDWLYYYAYRDFLWFLGRRFKAAAMLFVFTVSAVVHE 459
Dr_SOAT1  359 MVLCVFNSILPGVLVLFLAFFAFLHCWLNAFAEMLRFGDRMFYKDWWNSTSYANYYRTWNVVVHDWLYYYVYRDFLWMTQKRFRAVAMLVVFTVSAVVHE 458
Hs_DGAT1  319 IIERLLKLAVPNHLIWLIFFYWLFHSCLNAVAELMQFGDREFYRDWWNSESVTYFWQNWNIPVHKWCIRHFYKPMLRRGSSKW--MARTGVFLASAFFHE 416

                       M8                           M9
Hs_SOAT1  462 YALAVCLSFFYPVLFVLFMFFGMA----FNFIVNDSRKKPIWNVLMWTSLFLGNGVLLCFYSQEWYARQH-CPLKNPTFLDYVRPRSWTC--RYVF---- 550
Hs_SOAT2  436 YIFCFVLGFFYPVMLILFLVIGGM----LNFMMHDQRTGPAWNVLMWTMLFLGQGIQVSLYCQEWYARRH-CPLPQATFWGLVTPRSWSC--HT------ 522
Gg_SOAT1  472 YVLSICFGFFYPVLFCLFMCFGML----FNFILNDRRKGPIWNVIMWTSLFLGQGVIICLYSQEWYARQY-CPAENPAFLDYLKPRSWSC--HVQM---- 560
Xl_SOAT1  460 YALGICFGFFYPVLFILFMCFGML----FNFILHDRRRGPIWNVIMWTSLFLGQGVLICLYSQEWYAQRH-CPINNPAFTDFLKPRSWTCRRHHED---- 550
Dr_SOAT1  459 YVLAICFGFFYPVMFCLFMCFGMV----FNFVLHDRRKGPIWNVIMWTSLFLGQGVMICLYSQEWYAQRY-CPIEEPSLIDLLKPRSWTCYPQTNAAFDS 553
Hs_DGAT1  417 YLVSVPLR-----MFRLWAFTGMMAQIPLAWFVGRFFQGNYGNAAVWLSLIIGQPIAVLMYVHDYYVLNYEAPAAEA---------------------- 488
```

b

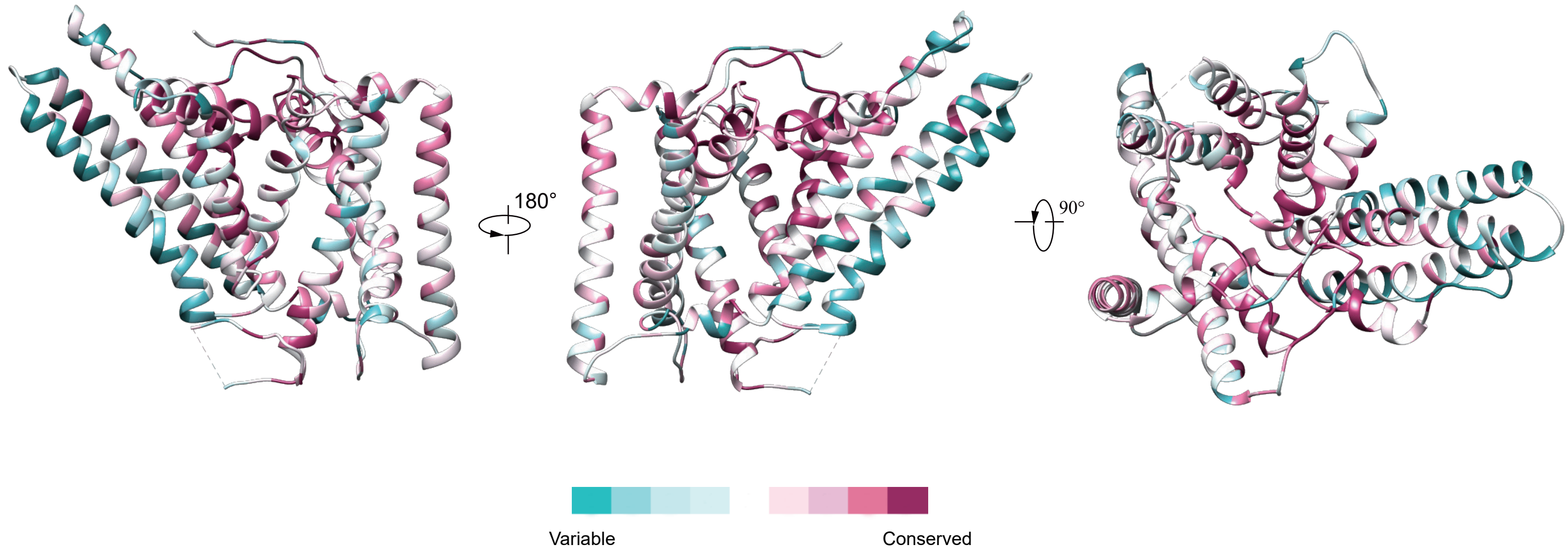

Supplementary Figure 7 | Sequence alignments of HsSOAT1, HsSOAT2, GgSOAT1, XlSOAT1, DrSOAT1 and HsDGAT1. a, The secondary structure elements are shown above the sequences (α-helices as cylinders, loops as lines and unmodeled residues as dashed lines). Conserved and highly conserved residues are highlighted in gray. Cylinders are colored in rainbow colors according to Fig. **3d**. The active site H460 is boxed in red. Two cysteines forming the disulfide bond in the ER lumen are boxed in blue. Residues that interact with the putative sterol-like molecule are boxed in green. Hs: homo sapiens, Gg: Gallus gallus, Xl: Xenopus laevis, Dr: Danio rerio. **b**, Structural conservation was analyzed using ConSurf server, based on sequence alignment of SOAT1 from 94 species using Clustal Omega, only single subunit of hSOAT1 is shown for clarity.

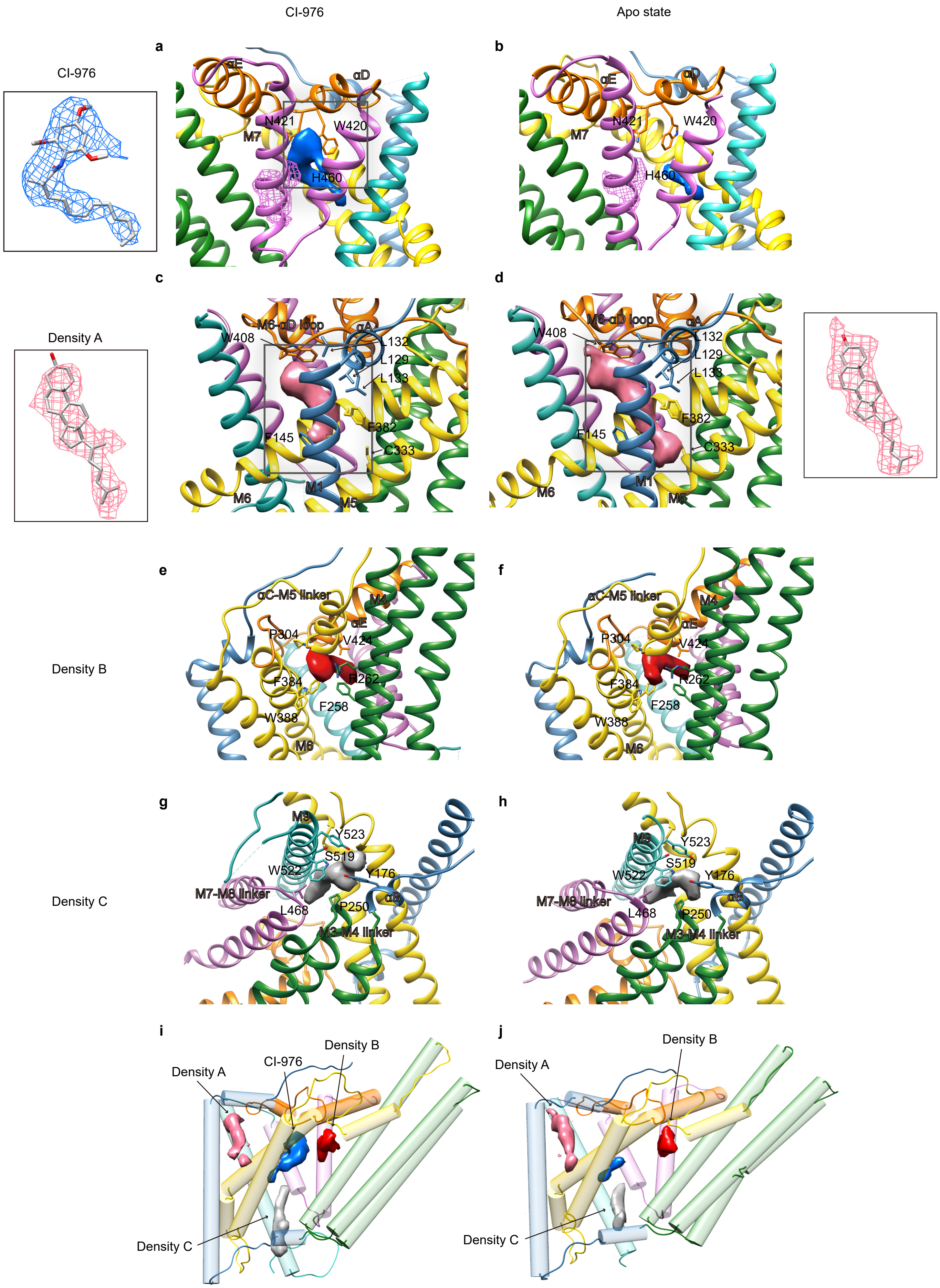

Supplementary Figure 8 | Electron density maps of bound ligands. a-b, Local EM densities inside the catalytic chamber in hSOAT1 dimer maps in complex with CI-976 (**a**) and in apo state (**b**). The inhibitor CI-976 density in (**a**) is shown as blue surface. The weak residual density in the BiSAS map is also shown as blue surface. The density of H460 side chains is shown in pink meshes at the same contour level as the ligand density in blue. Maps were further sharpened at -50 Å^2 by Coot for visualization. **c-d**, The sterol-like densities (density A) in the maps of hSOAT1 dimer in complex with CI-976 (**c**) and in apo state (**d**) are shown in pink. The close-up view of the density with a sterol-like molecule inside is shown in boxes. Maps were further sharpened at -120 Å^2 by Coot for visualization. **e-f**, The putative ligand densities (density B) in the maps of hSOAT1 dimer in complex with CI-976 (**e**) and in apo state (**f**) are shown in red. **g-h**, The putative ligand densities (density C) in the maps of hSOAT1 dimer in complex with CI-976 (**g**) and in apo state (**h**) are shown in grey. **i,** The non-protein density in the CI-976 map. **j,** The non-protein density in the apo state map.

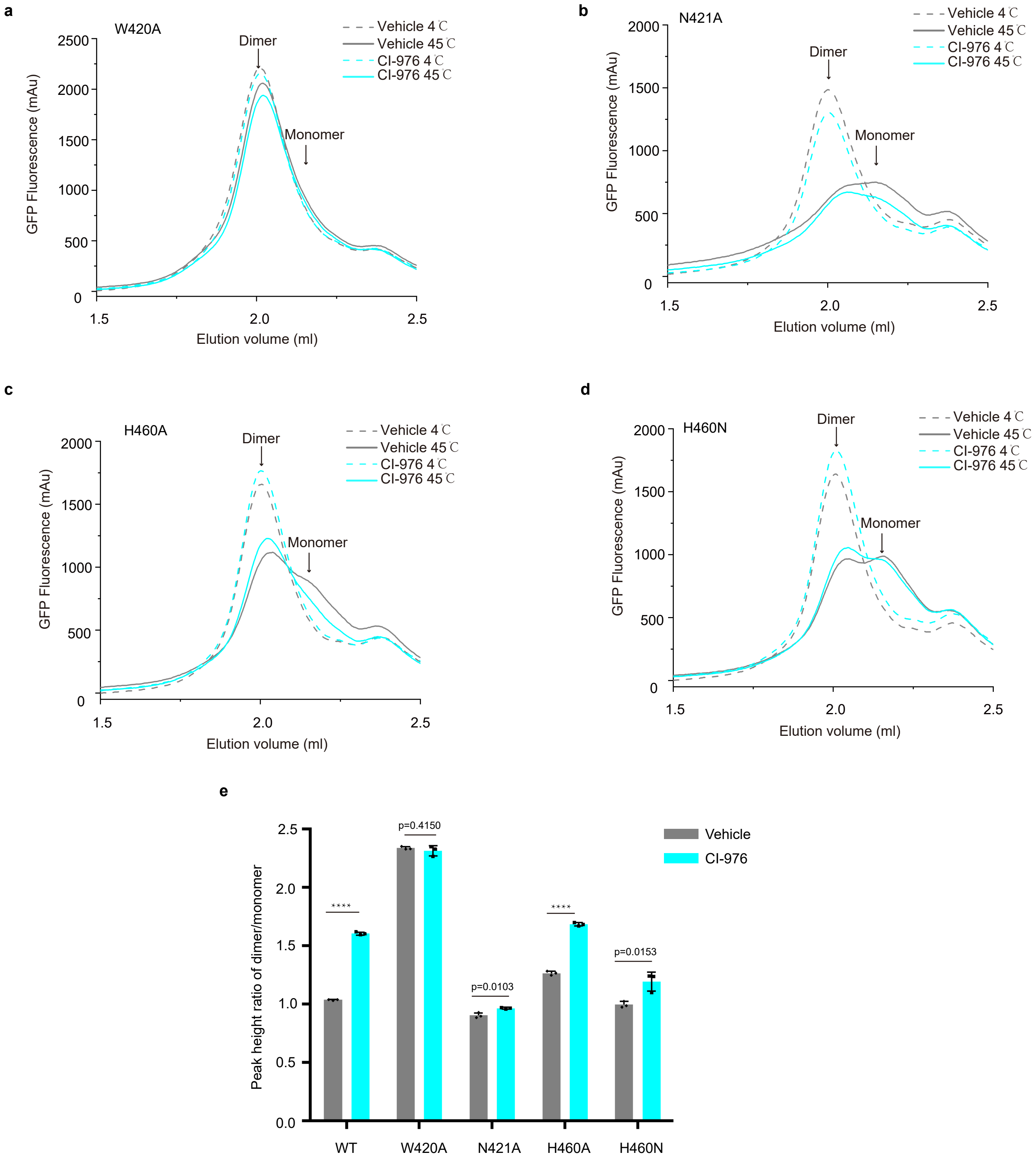

Supplementary Figure 9 | The thermostability tests of hSOAT1 dimer and mutants. a-d, Representative FSEC traces of N terminal GFP tagged SOAT1 dimer W420(**a**), N421A(**b**), H460A(**c**) and H460N (**d**) mutants at 4°C (dashed lines) or 45°C (solid lines) in the presence of 100 µM CI-976 (cyan) or DMSO alone (vehicle, gray). The elution positions of SOAT1 dimer and putative monomer are labeled by arrows. **e,** The peak height ratio of the dimer/monomer in the presence of DMSO alone (vehicle, gray) or 100 µM CI-976 (cyan) for various hSOAT1 dimer mutants (Data are shown as means± standard deviations, n=3 biologically independent samples, $p<0.0001$ for WT, $p=0.4150$ for W420A, $p=0.0103$ for N421A, $p<0.0001$ for H460A, $p=0.0153$ for H460N respectively by two-tailed paired t-tests).

a

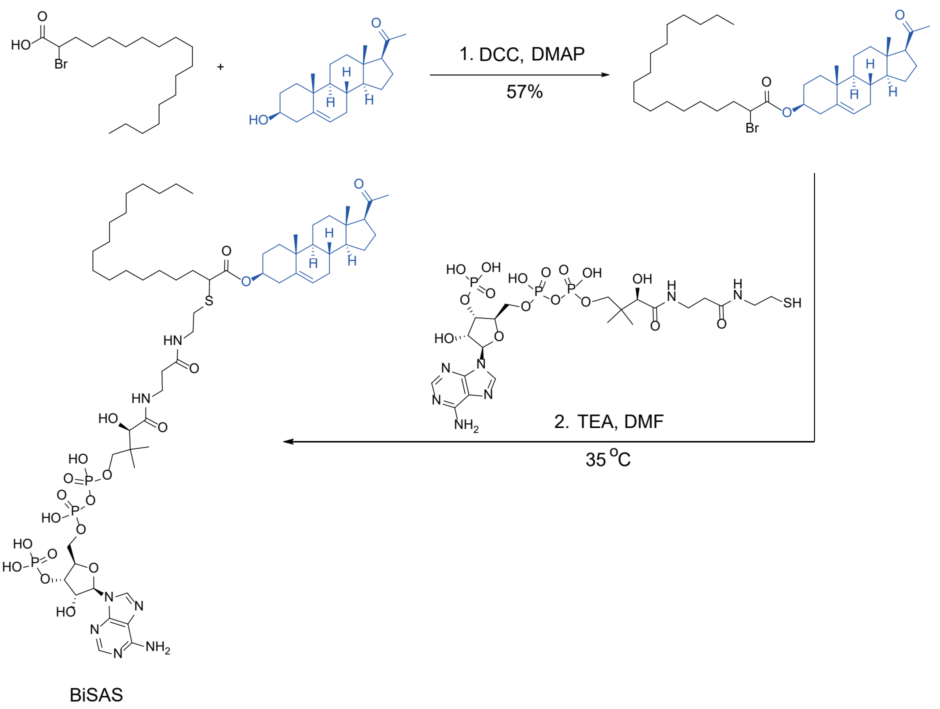

b

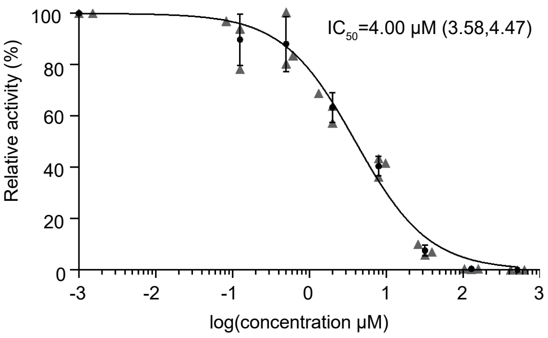

Supplementary Figure 10 | The chemical synthesis of BiSAS. a, Design and synthesis of BiSAS. **b**, Dose-dependent inhibition curve of hSOAT1 tetramer by BiSAS (The first data point is an artificial point. Data are shown as means ± standard deviations, n = 3 biologically independent samples).

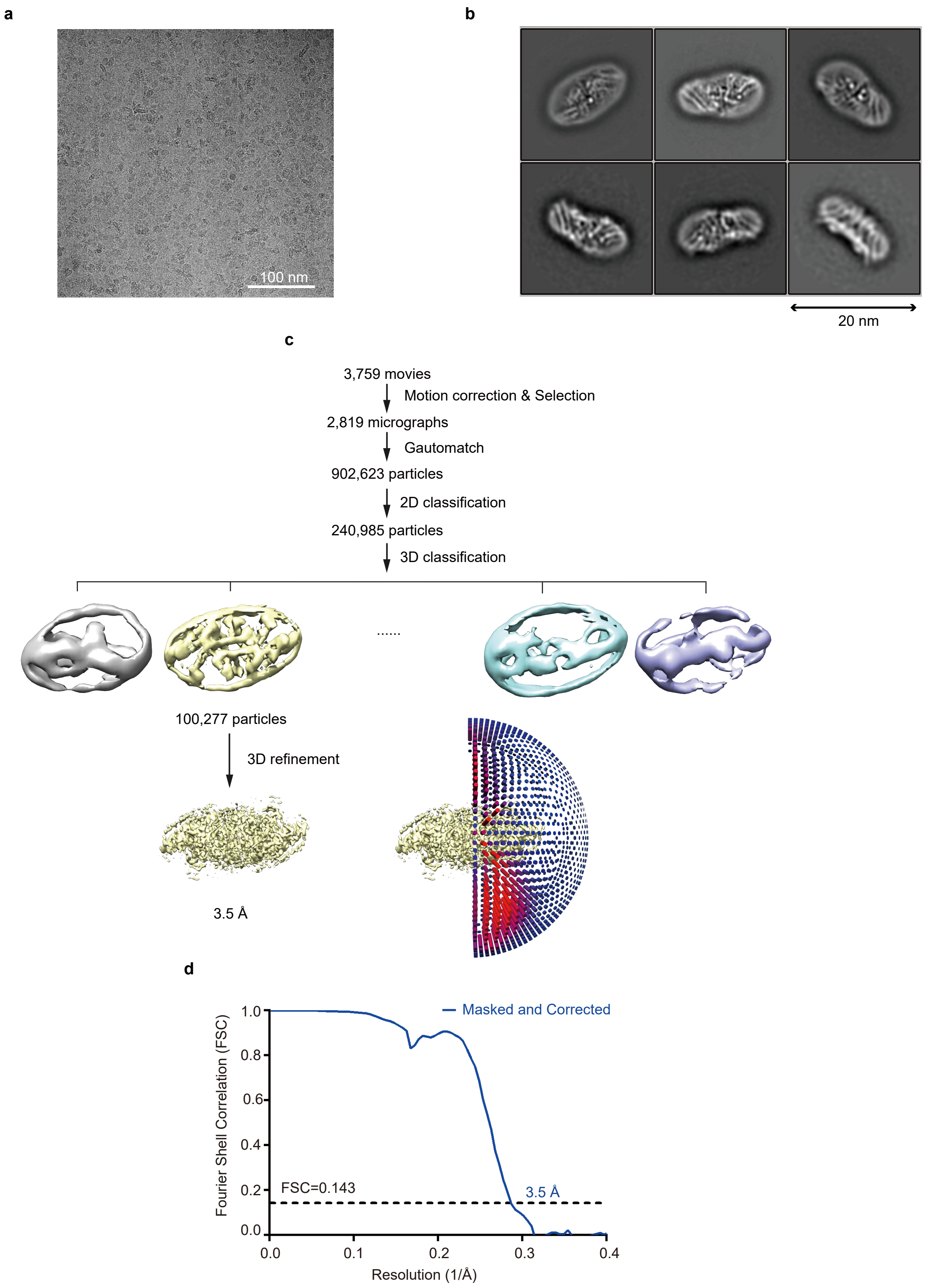

Supplementary Figure 11 | Cryo-EM image processing procedure of the hSOAT1 dimer in apo state. a, One representative raw micrograph of hSOAT1 dimer in apo state out of 3759 micrographs collected. **b**, Representative 2D class averages of the cryo-EM particles of hSOAT1 dimer in apo state. **c**, Flowchart of the image processing procedure for hSOAT1 dimer in apo state. **d**, Gold-standard Fourier shell correlation (FSC) curve of the final refined map for hSOAT1 dimer in apo state. Resolution estimation (3.5 Å) is based on the criterion of the gold-standard FSC cutoff at 0.143 in Relion.

a

b

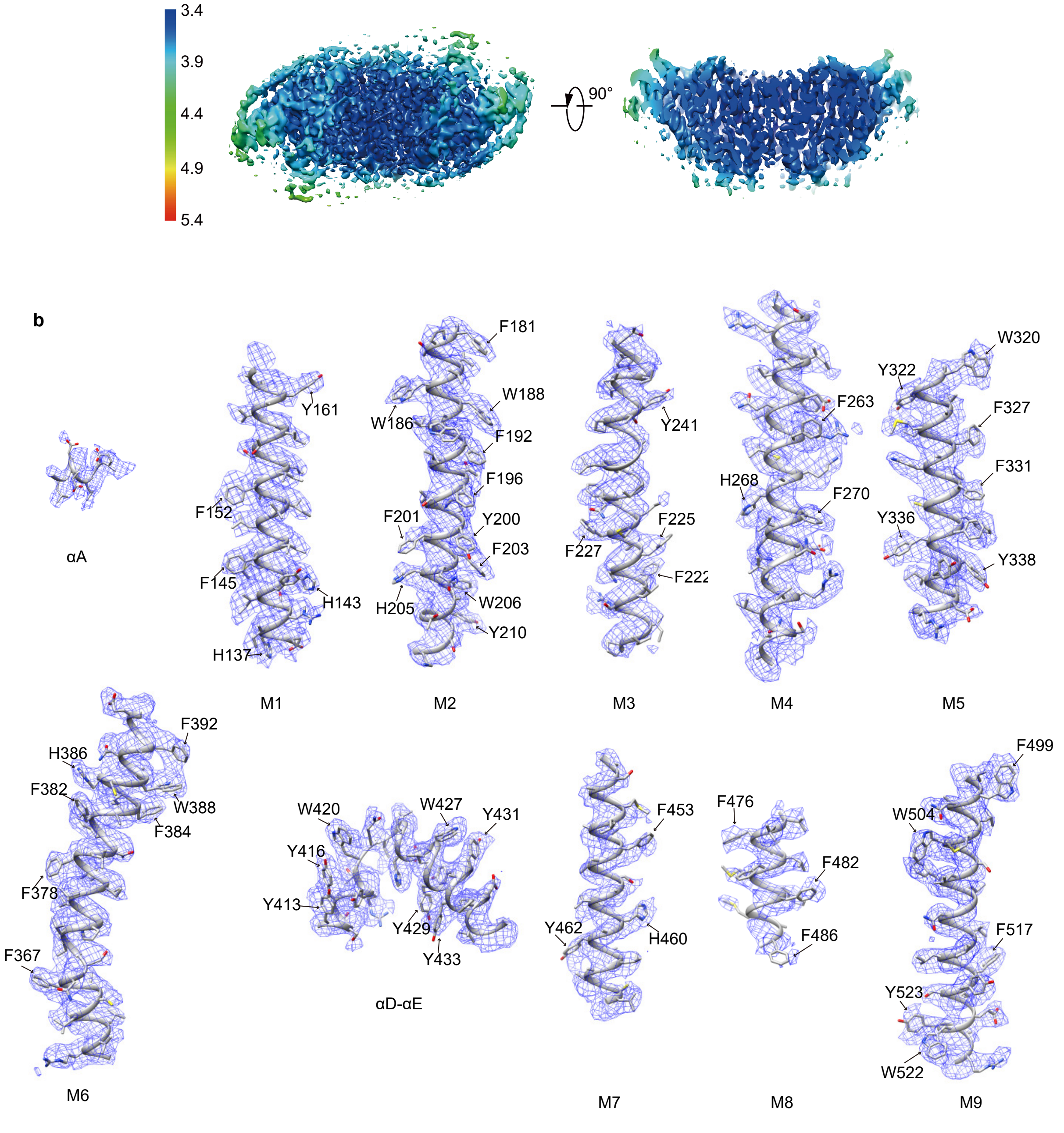

Supplementary Figure 12 | Electron density map of the hSOAT1 dimer in apo state. a, Top view and cut-away representations of the hSOAT1 dimer cryo-EM density map in apo state colored according to the local resolution estimation. **b**, EM density segments (blue mesh) of the 9 transmembrane helices (M1–M9), αA and αD-αE.

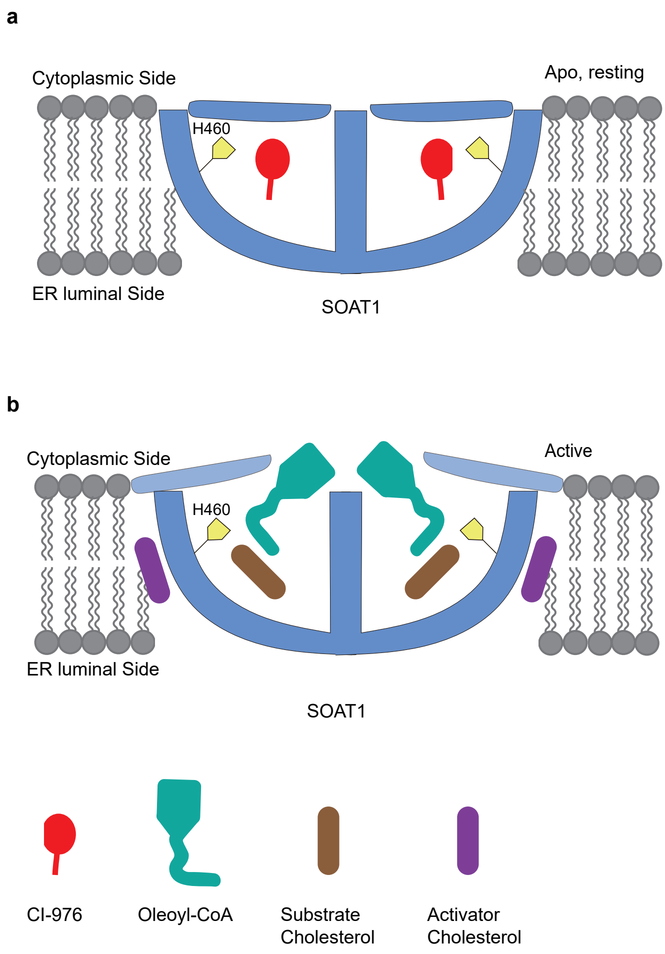

Supplementary Figure 13 | A working model for hSOAT1 activation. In the resting state, the putative catalytic residue H460 colored in yellow is less accessible to the acyl-CoA substrate. Inhibitor CI-976 in red blocks the catalytic center. The activator cholesterol in purple activates hSOAT1 by opening the lid of the reaction chamber to allow oleoyl-CoA in green and substrate cholesterol in brown to bind. For simplicity, only one SOAT1 dimer is shown.

Supplementary Table. 1
Cryo-EM data collection, refinement and validation statistics

	hSOAT1 Oval Tetramer	hSOAT1 Rhombic Tetramer	hSOAT1 dimer with CI-976	hSOAT1 apo dimer
PDB ID			6L47	6L48
EMDB ID	EMD-0829	EMD-0830	EMD-0831	EMD-0832
**Data collection and processing**				
Magnification	130,000 ×		130,000 ×	130,000 ×
Voltage (kV)	300		300	300
Electron exposure (e–/Å^2)	48		48	48
Defocus range (μm)	-1.5 to -2.0		-1.5 to -2.0	-1.5 to -2.0
Pixel size (Å)	1.045		1.045	1.045
Symmetry imposed	*C2*		*C2*	*C2*
Initial particle images (no.)	210,617		1,474,181	902,623
Final particle images (no.)	21,985	13,703	222,018	100,227
Map resolution (Å)	8.2	7.6	3.5	3.5
FSC threshold	0.143	0.143	0.143	0.143
Map resolution range (Å)	250-8.2	250-7.6	250-3.5	250-3.5
**Refinement**				
Initial model used (PDB code)			*De novo*	6L47
Model resolution (Å)			3.7	3.5
FSC threshold			0.143	0.143
Model resolution range (Å)			250-3.7	250-3.5
Map sharpening *B* factor (Å^2)			-90	-160
Model composition				
Non-hydrogen atoms			7,000	6,528
Protein residues			832	794
Ligands			4	2
B factors (Å^2)				
Protein			108.56	76.06
Ligand			110.17	36.21
R.m.s. deviations				
Bond lengths (Å)			0.003	0.003
Bond angles (°)			0.789	0.807
Validation				
MolProbity score			2.64	2.26
Clashscore			14.65	10.10
Poor rotamers (%)			3.34	2.45
Ramachandran plot				
Favored (%)			89.27	93.35
Allowed (%)			10.73	6.65
Disallowed (%)			0.00	0.00

Supplementary Table. 2
Primers used in this study

Primer Name	Sequence
hSOAT1-FL-F	ccaaggccctGAATTCGGATCC
hSOAT1-FL-R	CTCGAGcaccaccacc
hSOAT1Dimer-F	caaggccctgaattcATGAAGGAAGTTGGCAGTCAC
H460A_F	CTGTAGTAGCCGAATATGCCTTG
H460A_R	CATATTCGGCTACTACAGCAGATAC
H460N_F	GCTGTAGTAAACGAATATGCCTTG
H460N_R	CATATTCGTTTACTACAGCAGATAC
W420A-F	AGAACCGCCAATGTGGTGG
W420A-R	ACATTGGCGGTTCTATAATAG
N421-F	AACCTGGGCCGTGGTGGTCC
N421-R	CACCACGGCCCAGGTTCTAT
A147F-F	TATCACATGTTTATTTTCCTCCTCAT
A147F-R	AAAGAGAATGAGGAGGAAAATAAACA
L151W-F	TATTGCCCTCCTCATTTGGTTTATCCTC
L151W-R	GTGTGCTGAGGATAAACCAAATGAGGAG
L159W-F	CCTCAGCACACTTGTATGGGATTACATT
L159W-R	CTTCATCAATGTAATCCCATACAAGTG
T140R-F	AGTGGACCACATCAGAAGAATATATCAC
T140R-R	TAAACATGTGATATATTCTTCTGATGTG
Gg_SOAT1-F	CcaaggccctgaattcATGGCAGGTGAAGACTGTGTAAG
Gg_SOAT1-R	GgtggtggtgctcgagTTACATCTGCACGTGACATGAC
Xl_SOAT1-F	CcaaggccctgaattcATGTCTGATGAAGAGGGCCGC
Xl_SOAT1-R	GgtggtggtgctcgagCTAATCTTCATGATGGCGTC
Dr_SOAT1-F	CcaaggccctgaattcATGGTGAATGAGGGTGCTGG
Dr_SOAT1-R	GgtggtggtgctcgagTCAGTGAGAGTCAAAAGCAG
hSOAT2-F	ccaaggccct GAATTC ATGGAGCCAGGCGGG
hSOAT2-R	gtggtggtgCTCGAG CTAGGTATGGCAGGA
