# Supplementary material for: Structural insights into the inhibition mechanism of human sterol O-acyltransferase 1 by a competitive inhibitor
Source: Nat Commun. 2020 May 18;11:2478. doi: 10.1038/s41467-020-16288-4 (PMC7234994; doi:10.1038/s41467-020-16288-4)
Supplement: Supplementary file 3 — Reporting Summary [file 41467_2020_16288_MOESM3_ESM.pdf]

nature research

Corresponding author(s): Lei Chen

Last updated by author(s): 04/17/2020

Reporting Summary

Nature Research wishes to improve the reproducibility of the work that we publish. This form provides structure for consistency and transparency in reporting. For further information on Nature Research policies, see Authors & Referees and the Editorial Policy Checklist.

Statistics

For all statistical analyses, confirm that the following items are present in the figure legend, table legend, main text, or Methods section.

n/a	Confirmed	
☐	☒	The exact sample size (n) for each experimental group/condition, given as a discrete number and unit of measurement
☐	☒	A statement on whether measurements were taken from distinct samples or whether the same sample was measured repeatedly
☐	☒	The statistical test(s) used AND whether they are one- or two-sided<br>*Only common tests should be described solely by name; describe more complex techniques in the Methods section.*
☒	☐	A description of all covariates tested
☐	☒	A description of any assumptions or corrections, such as tests of normality and adjustment for multiple comparisons
☐	☒	A full description of the statistical parameters including central tendency (e.g. means) or other basic estimates (e.g. regression coefficient) AND variation (e.g. standard deviation) or associated estimates of uncertainty (e.g. confidence intervals)
☐	☒	For null hypothesis testing, the test statistic (e.g. F, t, r) with confidence intervals, effect sizes, degrees of freedom and P value noted<br>*Give P values as exact values whenever suitable.*
☒	☐	For Bayesian analysis, information on the choice of priors and Markov chain Monte Carlo settings
☒	☐	For hierarchical and complex designs, identification of the appropriate level for tests and full reporting of outcomes
☒	☐	Estimates of effect sizes (e.g. Cohen's d, Pearson's r), indicating how they were calculated

Our web collection on statistics for biologists contains articles on many of the points above.

Software and code

Policy information about availability of computer code

Data collection	SerialEM 3.6.11
Data analysis	MotionCor2, GCTF-1.0.6, Gautomatch-0.53, RELION2.0, RELION 3.0, cisTEM-1.0.0, cryoSPARC-0.6.5, PHENIX-1.10.1-2155, Coot-0.8.6, UCSF Chimera-1.10, Pymol-1.7.0.5, GraphPad Prism 6, Microsoft Excel , Resmap-1.1.4

For manuscripts utilizing custom algorithms or software that are central to the research but not yet described in published literature, software must be made available to editors/reviewers. We strongly encourage code deposition in a community repository (e.g. GitHub). See the Nature Research guidelines for submitting code & software for further information.

Data

Policy information about availability of data

All manuscripts must include a data availability statement. This statement should provide the following information, where applicable:

- Accession codes, unique identifiers, or web links for publicly available datasets
- A list of figures that have associated raw data
- A description of any restrictions on data availability

The cryo-EM map of hSOAT1 tetramer in oval shape, hSOAT1 tetramer in rhombic shape, hSOAT1 dimer bound with CI-976 and in apo resting state have been deposited in the EMDB under ID codes EMD-0829, EMD-0830, EMD-0831 and EMD-0832. The atomic coordinate of hSOAT1 dimer bound with CI-976 and in apo resting state have been deposited in the PDB under ID codes 6L47 and 6L48.

Field-specific reporting

Please select the one below that is the best fit for your research. If you are not sure, read the appropriate sections before making your selection.

☒ Life sciences ☐ Behavioural & social sciences ☐ Ecological, evolutionary & environmental sciences

For a reference copy of the document with all sections, see nature.com/documents/nr-reporting-summary-flat.pdf

Life sciences study design

All studies must disclose on these points even when the disclosure is negative.

Sample size	No predetermination of sample size was performed. Sufficient cryo-EM data were collected to achieve adequate map resolutions for model building (). The enzymatic activity assay experiments were performed with three biological replicates. The sample size was based on previous studies in the field (PMID: 8798656) and clearly indicated in the legends.
Data exclusions	Cryo-EM micrographs with ice or ethane contamination, empty carbon, and poor CTF fit (> 5 Å) were excluded manually. Particles belonging to bad classes were discarded and the data processing flowchart were summarized in Extended Data Figures. These criteria were pre-established and the procedure is a common practise in cryo-EM image analysis. No data was excluded in functional studies.
Replication	All attempts at replication were successful according to the detailed protocol described in the methods section. The numbers of replication were described in figure legends.
Randomization	For cryo-EM 3D refinement, all particles were randomly split into two groups. Randomization is not applicable to other experiments because no covariates were involved.
Blinding	The investigators were blinded to group allocation during cryo-EM data collection and analysis. No blinding is performed in other experiments because there is no comparison inside each experimental group.

Reporting for specific materials, systems and methods

We require information from authors about some types of materials, experimental systems and methods used in many studies. Here, indicate whether each material, system or method listed is relevant to your study. If you are not sure if a list item applies to your research, read the appropriate section before selecting a response.

Materials & experimental systems

n/a	Involved in the study	
☐	☒	Antibodies
☐	☒	Eukaryotic cell lines
☒	☐	Palaeontology
☒	☐	Animals and other organisms
☒	☐	Human research participants
☒	☐	Clinical data

Methods

n/a	Involved in the study	
☒	☐	ChIP-seq
☒	☐	Flow cytometry
☒	☐	MRI-based neuroimaging

Antibodies

Antibodies used	Mouse monoclonal antibody against human and mouse vinculin was purchased from Millipore, catalog # 05-386, with 5000-fold dilution. Antibody against human and mouse ACAT1/SOAT1:DM10 was isolated and affinity purified in Chang Lab, 0.1 µg/ml was used.
Validation	Certificate of analysis from Miliipore: Anti-Vinculin, clone V284 (mouse monoclonal IgG1) - 2239859. DM10 antibody was validated in PMID: 7493995.

Eukaryotic cell lines

Policy information about cell lines

Cell line source(s)	FreeStyle 293F and Sf9 were from Thermo Fisher Scientific. WT CHO cell was from ATCC. AC29 cell line is a mutant CHO cell line and was generated and kept in the Chang lab. HEK293S cell line is a stable cell line and was generated and kindly provided by Professor H Gobind Khorana at MIT.
Authentication	None of the cell line used was authenticated.
Mycoplasma contamination	All cell lines were tested negative for mycoplasma contamination.
Commonly misidentified lines (See ICLAC register)	No commonly misidentified cell lines were used.
