# Supplementary material for: Structural insights into the inhibition mechanism of human sterol O-acyltransferase 1 by a competitive inhibitor
Source: Nat Commun. 2020 May 18;11:2478. doi: 10.1038/s41467-020-16288-4 (PMC7234994; doi:10.1038/s41467-020-16288-4)
Supplement: Supplementary file 2 — Peer Review File [file 41467_2020_16288_MOESM2_ESM.pdf]

Editorial Note: This manuscript has been previously reviewed at another journal that is not operating a transparent peer review scheme. This document only contains reviewer comments and rebuttal letters for versions considered at Nature Communications.

REVIEWERS' COMMENTS:

Reviewer #1 (Remarks to the Author):

The authors have considerably strengthened the functional analysis and the paper in general. I have no further concerns.

Reviewer #2 (Remarks to the Author):

The authors have addressed my remarks adequately by introducing new data and analyses.
As a result the manuscript has improved significantly. It is now a timely and comprehensive piece of work that will be valuable to the diverse research communities studying SOAT-family proteins and their therapeutic targeting.
